# Supplementary material for: Engineering new metabolic pathways in isolated cells for the degradation of guanidinoacetic acid and simultaneous production of creatine
Source: Mol Ther Methods Clin Dev. 2022 Feb 22;25:26–40. doi: 10.1016/j.omtm.2022.02.007 (PMC8917272; doi:10.1016/j.omtm.2022.02.007)
Supplement: Document S1. Supplemental material and methods, Figures S1–S4 and Table S1 [file mmc1.pdf]

OMTM, Volume *25*

Supplemental information

Engineering new metabolic pathways in isolated cells for the degradation of guanidinoacetic acid and simultaneous production of creatine

Marzia Bianchi, Luigia Rossi, Francesca Pierigè, Pietro De Angeli, Mattia Paolo Aliano, Claudia Carducci, Emanuele Di Carlo, Tiziana Pascucci, Francesca Nardecchia, Vincenzo Leuzzi, and Mauro Magnani

Supplemental Information

Cloning strategies to optimize recombinant human GAMT expression

In a first attempt, the GAMT coding sequence was cloned in a pET45b expression plasmid directly fused to the His-tag coding sequence. The expression vector has been transformed in the BL21(DE3) *E. coli* strain and the clone showed a good expression of the N-terminal His-tagged recombinant protein, although the most part was in the insoluble fraction (Figure S1A). Different induction protocols were tested, changing the temperature and/or the length of induction, the cell density at which GAMT expression was induced, but in no case any improvement was observed (not shown). To overcome the drawbacks of the His-tag fusion strategy and to finalize the expression for therapeutic use, we designed and prepared two further constructs, using both pET45b and pET22b expression vectors in which the GAMT insert was cloned in such a way to produce the amino acid sequence of GAMT without any extra part (NO-tag GAMT). Unfortunately, the GAMT protein was expressed in a very low amount (or not expressed at all) (Figure S1B-C) and this result was not overcome by using different experimental conditions (different expression host, use of synthetic media). Moreover, a low growth rate was observed upon IPTG induction, which opened the hypothesis that GAMT might be toxic for bacteria, considering that it uses as substrate SAM that is very important in different endogenous pathways, like bacterial cell division (Newman et al., 1998). Following the hypothesis of GAMT toxicity for the host expressing cells, a construct referred to as pET22b-pelB-GAMT was generated, to target GAMT into the periplasmic space. The induction tests revealed that GAMT was expressed in the insoluble fraction as inclusion bodies (Figure S1D). Unfortunately, protein purification from the insoluble fraction and refolding requires too much time, difficult approaches and, moreover, the strategy used does not ensure that the pelB signal is completely removed. Then, a different expression strategy was attempted by cloning the GAMT CDS in the pET22b backbone in frame with the C-terminal His-tag provided in the cloning/expression region of the vector. However, this further expression construct (pET22b-GAMT-His-Tag) behaved like the pET22b-GAMT and pET45b-GAMT described above, with an undetectable recombinant GAMT expression (Figure S1E). Thus, unexpectedly, only the N-terminal Tag-based strategies allowed the expression of the recombinant enzyme.

Table S1. Primers used for cloning, site-directed mutagenesis and sequencing.

Fwd, forward; Rev, reverse. †Numbers indicate the 5' position of the sequencing primers, referring to the GAMT coding sequence (NCBI Reference Sequence: NM_000156.5) and to the MAT coding sequence (NCBI Reference Sequence: DQ083239.1). Restriction enzyme cutting sites in cloning primers are underlined. Nucleotide changes in the GAMT mutagenesis primer sequences are in bold and underlined and respective codons are highlighted in grey. Codon changes and relative amino acid substitutions are reported in the third column.

**NAME**	**SEQUENCES (5' to 3')**	**NOTES**
**Cloning primers**		
GAMT PmlI_Fwd	ACGGTACTTCACGTGATGAGCGCCCCCAGCGCGA	
UB-GAMT NcoI_Fwd	ACGGTACTTCCATGGGCCCCCAGCGCGACCCCC	
GAMT HindIII_Rev	GGATACCTAAAGCTTCAGCCTTTGGTCACCAGGGGCG	
MAT2A SacII_Fwd	CGTCTCCGCGGTGGAATGAACGGACAGCTCAACGGC	
MAT2A NotI_Rev	CGTCTGCGGCCGCTCAATATTTAAGCTTTTTGGGCACTTCCC	
**Mutagenesis primers**		**Codon change (aa change)**
UB-GAMT (L37I)	CACGCACCTGCGCATC**A**T**T**GGCAAGCCGGTGATGG	CTG → ATT (Leu→Ile)
UB-GAMT (G38A)	ACCTGCGCATCCTGG**C**CAAGCCGGTGATGGAGC	GGC → GCC (Gly→Ala)
UB-GAMT (L37I - G38A)	CACGCACCTGCGCATC**A**T**T**G**C**CAAGCCGGTGATGGAGC	CTG → ATT (Leu→Ile) GGC → GCC (Gly→Ala)
UB-GAMT (L37M)	ACGCACCTGCGCATC**A**TGGGCAAGCCGGTGATGG	CTG → ATG (Leu→Met)
UB-GAMT (I187Q)	GTACTCAGACATCACC**CAG**ATGTTTGAGGAGACGCAGGTG	ATC → CAG (Ile→Gln)

UB-GAMT (A26N)	GGGCGGCGCCCGCG**AA**CTACGACGCAGCGGAC	GCC → AAC (Ala→Asn)
UB-GAMT (V215Q)	GGAGGTGATGGCGCTG**CAG**CCACCGGCCGACTGCC	GTC → CAG (Val→Gln)
**Sequencing primers**		
GAMT 7†_Fwd	GCCCCCAGCGCGACCCCCATCT	
GAMT 312†_Fwd	ACGGCAGACACACAAGGTCATCC	
GAMT 711†_Rev	TCAGCCTTTGGTCACCAGGGGC	
MAT2A 100†_Fwd	GTGACCAAATCAGTGATGCTGTCC	
MAT2A 398†_Fwd	AGACCAGGGCTTAATGTTTGGC	
MAT2A 799†_Fwd	TTGTGGACACTTATGGCGGTTG	
T7 promoter	TAATACGACTCACTATAGGG	
T7 terminator	GCTAGTTATTGCTCAGCGG	

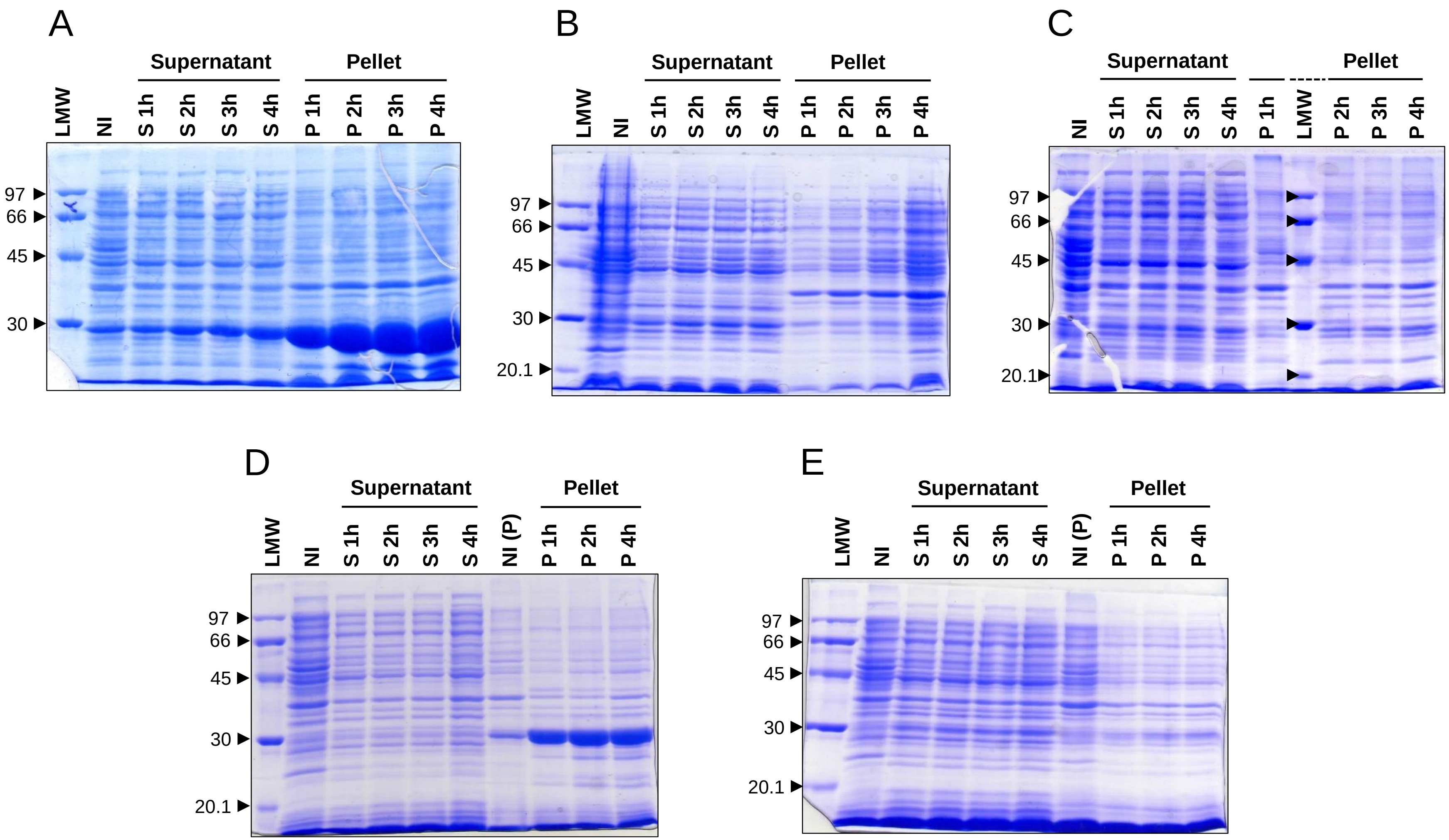

Figure S1: Cloning strategies to optimize recombinant human GAMT expression. [A] pET45b/His-GAMT; [B] pET45b/GAMT (NO tag); [C] pET22b/GAMT (NO tag); [D] pET22b/pelB-GAMT; [E] pET22b/GAMT-His. SDS-PAGE of representative time-course induction experiments with the different expression constructs. Supernatant (S) and Pellet (P) fractions are compared; NI is the not-induced sample. LMW, low molecular weight protein standards.

A

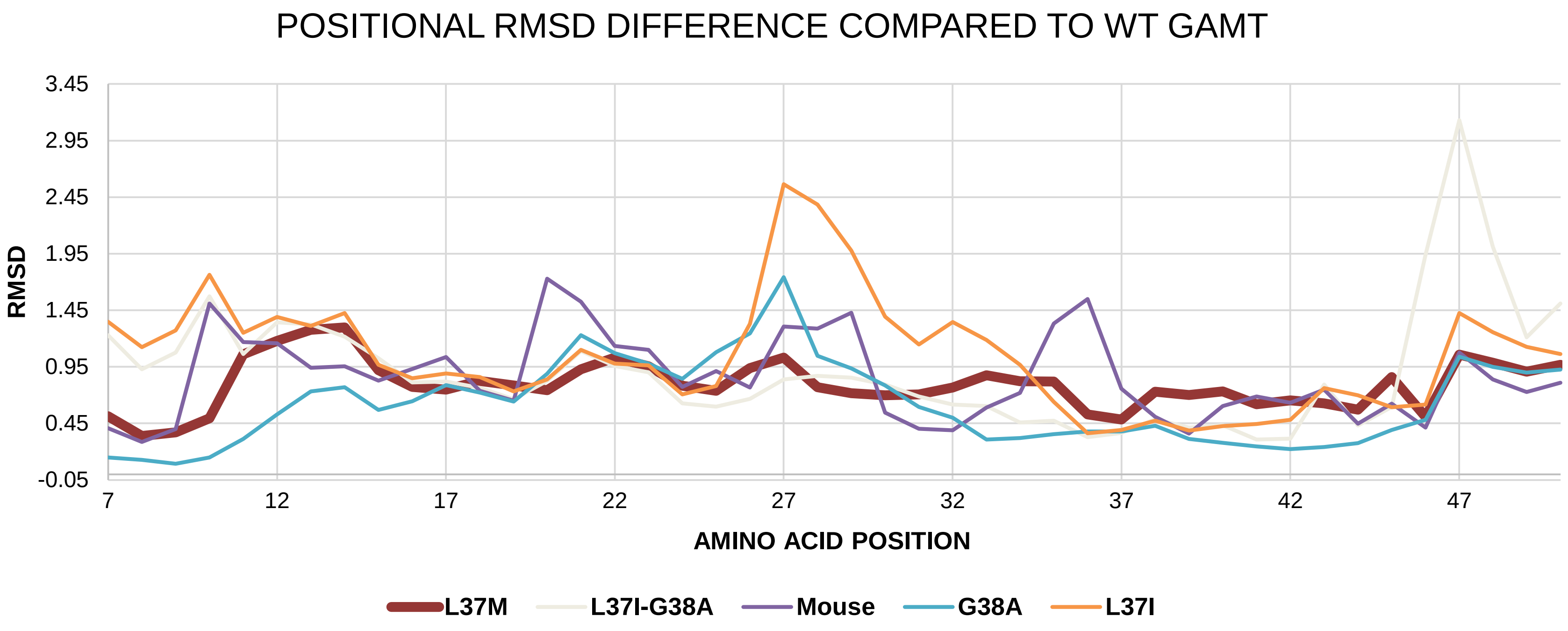

B

	Mean RMSD
**L37I-G38A**	0.93
**L37M**	0.81
**Mouse**	0.88
**G37A**	0.64
**L37I**	1.07

Figure S2: In silico studies of GAMT protein flexibility. [A] Graph plotting the predicted root-mean-square deviation (RMSD) difference of the GAMT mutants compared to wild type, against protein chain position for the first 47 amino acid residues. [B] Mean RMSD difference of the GAMT mutants compared to wild type GAMT for the first 47 amino acid residues.

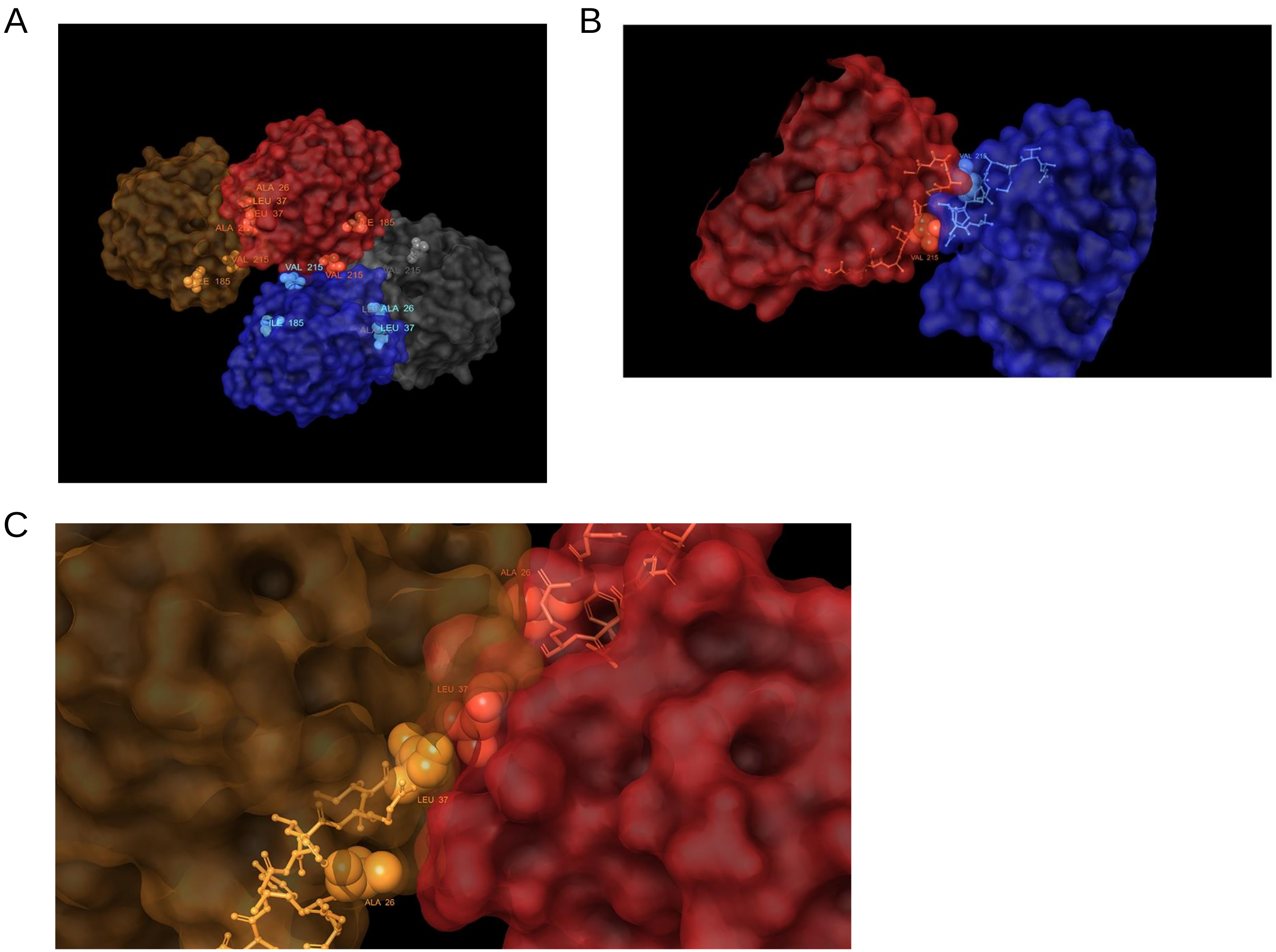

Figure S3: In silico studies of GAMT intermolecular interactions. [A] GAMT homotetramer as in 3orh PDB. The mutagenized amino acid residues are highlighted. [B] GAMT homodimer interface involving Val215 residues. [C] GAMT homodimer interface involving Ala26 and Leu36 residues.

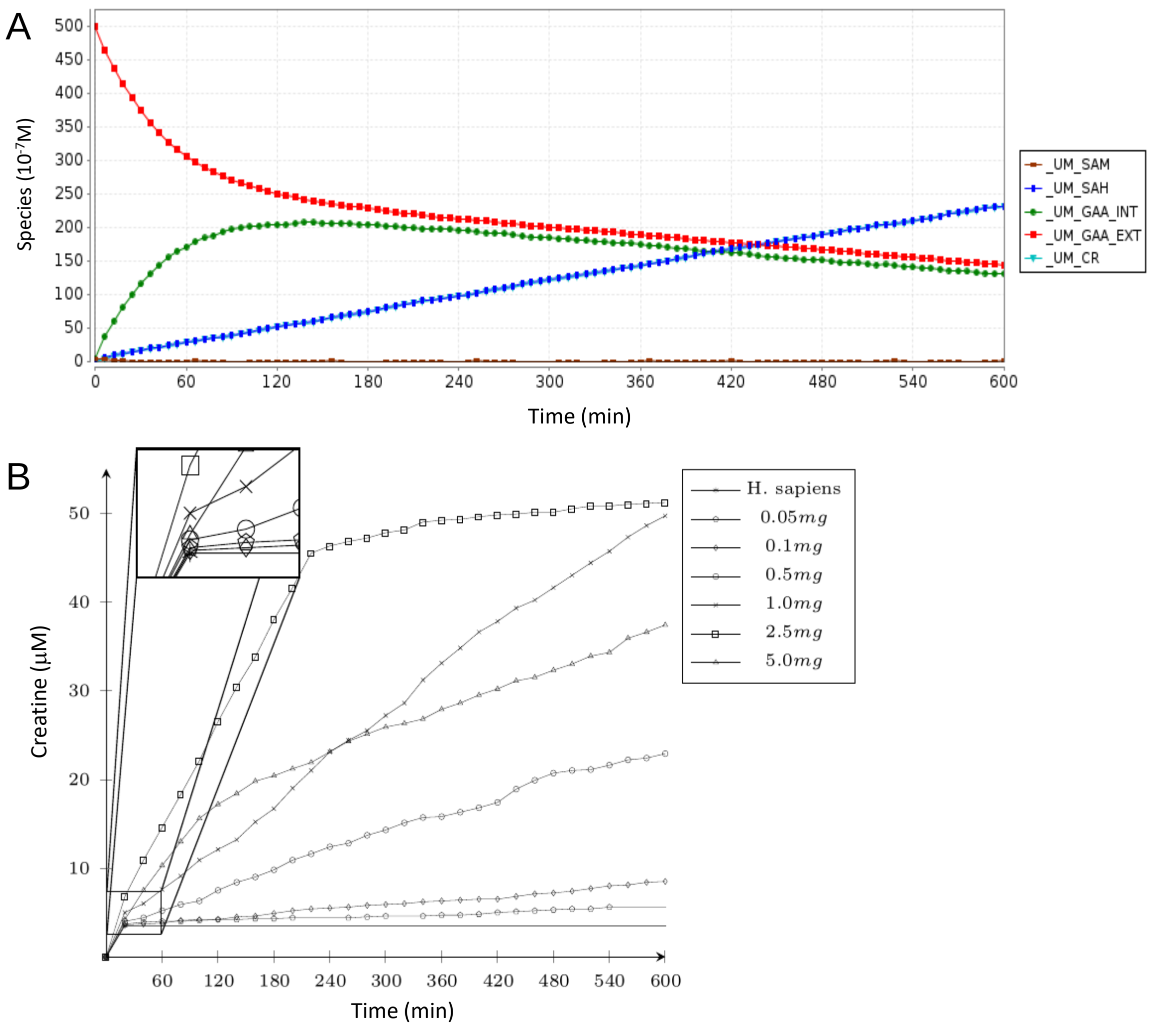

Figure S4: **Metabolic modeling of GAMT-loaded RBCs.** [A] Behaviour of RBCs engineered with 1 mg of recombinant GAMT cloned from *E. coli*. The Y axis indicates the concentration (10^{-7}M) of the different species. [B] Bio-PEPA model of Creatine synthesis pathway by engineered RBCs loaded with a fixed amount of GAMT (1mg) and different amounts of recombinant MAT (as detailed in the inset). Each model was simulated for 10 hours (X axis), with a sampling every 10 minutes.

REFERENCES

Newman, E.B., Budman, L.I., Chan, E.C., Greene, R.C., Lin, R.T., Woldringh, C.L., D'Ari, R. (1998). Lack of S-adenosylmethionine results in a cell division defect in Escherichia coli. J Bacteriol. 180, 3614-3619.
