# Supplementary material for: Engineering new metabolic pathways in isolated cells for the degradation of guanidinoacetic acid and simultaneous production of creatine
Source: Mol Ther Methods Clin Dev. 2022 Feb 22;25:26–40. doi: 10.1016/j.omtm.2022.02.007 (PMC8917272; doi:10.1016/j.omtm.2022.02.007)
Supplement: Document S2. Article plus supplemental information [file mmc2.pdf]

Molecular Therapy
Methods & Clinical Development
Original Article

Engineering new metabolic pathways in isolated cells for the degradation of guanidinoacetic acid and simultaneous production of creatine

Marzia Bianchi,[1,8] Luigia Rossi,[1,2,8] Francesca Pierigè,[1] Pietro De Angeli,[3] Mattia Paolo Aliano,[1] Claudia Carducci,[4] Emanuele Di Carlo,[4] Tiziana Pascucci,[5,6] Francesca Nardecchia,[7] Vincenzo Leuzzi,[7] and Mauro Magnani[1,2]

[1]Department of Biomolecular Sciences, University of Urbino Carlo Bo, 61029, Urbino, Italy; [2]EryDel, Via Antonio Meucci 3, 20091 Bresso, Milan, Italy; [3]Center for Ophthalmology, Institute for Ophthalmic Research, University of Tübingen, Tübingen, Germany; [4]Department of Experimental Medicine, Sapienza University, 00161 Rome, Italy; [5]Department of Psychology and "Daniel Bovet" Center, Sapienza University, 00184 Rome, Italy; [6]Istituto di Ricovero e Cura a Carattere Scientifico Fondazione Santa Lucia, 00142 Rome, Italy; [7]Division of Child Neurology and Psychiatry, Department of Human Neuroscience, Sapienza University, 00185 Rome, Italy

Here we report, for the first time, the engineering of human red blood cells (RBCs) with an entire metabolic pathway as a potential strategy to treat patients with guanidinoacetate methyltransferase (GAMT) deficiency, capable of reducing the high toxic levels of guanidinoacetate acid (GAA) and restoring proper creatine levels in blood and tissues. We first produced a recombinant form of native human GAMT without any tags to encapsulate into RBCs. Due to the poor solubility and stability features of the recombinant enzyme, both bioinformatics studies and extensive optimization work were performed to select a mutant GAMT enzyme, where only four critical residues were replaced, as a lead candidate. However, GAMT-loaded RBCs were ineffective in GAA consumption and creatine production because of the limiting intra-erythrocytic S-adenosyl methionine (SAM) content unable to support GAMT activity. Therefore, a recombinant form of human methionine adenosyl transferase (MAT) was developed. RBCs co-entrapped with both GAMT and MAT enzymes performed, *in vitro*, as a competent cellular bioreactor to remove GAA and produce creatine, fueled by physiological concentrations of methionine and the ATP generated by glycolysis. Our results highlight that metabolic engineering of RBCs is possible and represents proof of concept for the design of novel therapeutic approaches.

INTRODUCTION

Guanidinoacetate methyltransferase (GAMT; EC:2.1.1.2) deficiency (OMIM: 612736) was recognized as the first autosomal recessive inborn error of creatine metabolism in 1994 and is the most severe condition among the cerebral creatine deficiency syndromes (CCDSs).[1,2] GAMT deficiency is a rare disease, with an incidence of 1:550,000–1:2,640,000 newborns[3] and a prevalence of <1/1,000,000 (www.orpha.net). This enzyme catalyzes the last step of creatine synthesis, facilitating the transfer of a single methyl group from S-adenosyl methionine (SAM) to guanidinoacetate acid (GAA), forming creatine and S-adenosylhomocysteine (adoHcys). Creatine, a molecule of vital importance for the energy of the organism, is synthesized in the liver of all mammals, released into the blood, and captured by the tissues that require it.[1] At the biochemical level, the disease is characterized by extremely low levels of creatine in the tissues and bodily fluids (urine, plasma, and cerebrospinal fluid) and by the accumulation of its precursor GAA, a highly toxic molecule for the organism, especially for the brain.[2,4] GAMT defect phenotype is characterized by developmental delay/arrest, leading to severe intellectual disability with relevant language impairment,[2,5] developmental epileptic encephalopathy,[4,6] and movement disorders in about half of subjects.[3,7] A lifetime treatment aimed at restoring brain creatine and preventing GAA accumulation has been suggested and includes creatine supplementation, limited arginine intake with the diet, and ornithine and/or sodium benzoate supplementation. The treatment has proved to be effective in preventing the emergence of the GAMT-deficient phenotype in pre-symptomatic patients, while in late treated subjects improved the pharmacological control of the seizures.[8,9] To be successful, the therapy must be strictly followed, which can often lead to poor compliance of patients and their caregivers. Within this context, there is, therefore, an urgent need for novel therapeutic approaches that could enable an efficient treatment of subjects suffering from a deficit of creatine. An attractive and suitable option consists of removing the high levels of circulating GAA from blood and restoring normal levels of creatine in the brain throughout the administration of synthetic GAMT, the defective enzyme, in a cellular context capable of providing SAM as methyl donor. The simplest cell abundantly present in circulation is the erythrocyte (red blood cell [RBC]). Other authors and we have already reported that RBCs have unique properties that permit the encapsulation of recombinant enzymes without affecting their *in vivo* circulation and all main biochemical, immunological, and morphological properties.[10]

Received 13 November 2021; accepted 19 February 2022;
https://doi.org/10.1016/j.omtm.2022.02.007.

[8]These authors contributed equally.

Correspondence: Mauro Magnani, Department of Biomolecular Sciences, University of Urbino Carlo Bo, 61029 Urbino, Italy.
E-mail: mauro.magnani@uniurb.it

 © 2022 The Author(s).
This is an open access article under the CC BY license (http://creativecommons.org/licenses/by/4.0/).

Indeed, selected RBC constructs are in clinical development, as recently reported by Rossi et al.[10] Based on these premises, we have engineered human RBCs by the reconstruction of a convenient metabolic pathway that takes advantage of the cell membrane permeability to uptake plasma GAA to be converted into creatine that is later released into the circulatory system. In fact, creatine can be transported out of the cell by a creatine low-affinity transport, representing an exchange diffusion,[11] and, in any case, high creatine levels inside RBCs do not damage the RBCs themselves.[12] This approach requires the design and production of both enzymes, namely GAMT and methionine adenosyl transferase (MAT; EC:2.5.1.6), as described in this paper. In a similar way, mice erythrocytes have been engineered to be used as bioreactors to decrease ammonium concentration in blood.[13] However, to the best of our knowledge, for the first time, an entire metabolic pathway has been engineered into human RBCs. The whole metabolic pathway from GAA to creatine is operative in RBCs loaded with recombinant human GAMT and MAT, at different ratios, while physiological levels of methionine in human plasma can sustain the formation of creatine. It is envisaged that these metabolically engineered cells can be used as a medicament, in particular for the treatment of subjects suffering from a disease or condition caused and/or characterized by a deficit of creatine, like GAMT deficiency,[14] and methionine-dependent tumors.[15]

RESULTS

Cloning strategies to optimize recombinant human GAMT expression

Different expression strategies were explored to obtain a recombinant human GAMT, with the final aim to produce an enzyme suitable for therapeutic purposes. The GAMT coding sequence was initially cloned in a pET45b expression plasmid directly fused to the His-tag coding sequence and transformed in BL21(DE3) *Escherichia coli* strain. All attempts to optimize expression resulted in most of the protein being insoluble. Removal of the tag resulted in poor expression and the addition of a pelB signal sequence instead generated an insoluble product. Only the N-terminal tag-based strategies allowed the expression of the recombinant enzyme (further details in the supplemental information and Figure S1). Thus, starting from these results, a construct was designed and developed aimed to express an N-terminal tagged GAMT precursor and introduce a cleavage site to specifically remove the added tag. GAMT was cloned in the pET45b-UB plasmid, where the recombinant product, His-UB-GAMT, can be cleaved with a deubiquitylating enzyme, ubiquitin-specific protease 2 (USP2), resulting in the removal of the His-ubiquitin partner and releasing the natural GAMT protein (Figure 1A). This construct, named pET45b/His-UB-GAMT, allowed the expression of the protein at high levels, although mostly retained in the insoluble fraction (Figure 1B). The no-tag recombinant enzyme was obtained from the supernatant fraction by Ni Sepharose-affinity chromatography, performed before and after USP2 digestion (Figure 1C).

Kinetic activity and stability of native GAMT

The recombinant human GAMT no-tag showed a good affinity for its substrate GAA with K_M value of 0.057 ± 0.009 mM and a specific activity (S.A.) of 0.08 ± 0.02 μmol/min/mg. However, the enzyme did not show acceptable stability, especially when stored at 4°C. Figure 1E shows S.A. values of native GAMT, stored for different times (3 days and 1, 2, 3, 5, and 7 weeks) at different temperatures (4°C, −20°C, and −80°C). A rapid decrease of enzymatic activity was detected already after 3 days at 4°C (0.05 ± 0.009 μmol/min/mg versus 0.08 ± 0.02 μmol/min/mg at time 0) until to a complete disappearance after 2 weeks of storage. Aliquots stored at −20°C and −80°C have unchanged S.A. values, highlighting the effectiveness of the conservation process. These data are confirmed by the SDS-PAGE (Figure 1D), in which an evident thinning of the band corresponding to the intact enzyme can be observed at 4°C storage only.

Native GAMT engineering to increase protein stability and solubility

The pET45b/His-UB-GAMT construct underwent solubility and stability optimization before project scaling-up. Of note, no refolding of the protein from inclusion bodies was attempted because the GAMT no-tag purified from the soluble fraction already showed degradation (Figure 1D) and formation of visible precipitates over time. Based on personal experimental evidence consistent with the results reported by Komoto et al.[16] for rGAMT from rat liver, Leu37 and Gly38 residues show propensity to protease cleavage (Figure 1D). To increase the stability of recombinant GAMT (rGAMT), different site-directed mutagenesis targeting Leu37 and Gly38 residues were performed to generate three mutagenized expression constructs, carrying analogs of amino acid residues: hydrophobic amino acid substitution Leu37Ile (L37I), amino acid substitution of the small residue Gly38Ala (G38A), and the combination L37I-G38A (Figure 2A). All the mutants proved to be stable under different conditions (the stability of the double mutant L37I-G38A GAMT in both the precursor [His-UB-GAMT] and no-tag [GAMT] form is shown in Figures 2B and 2C); however, the no-tag single mutant L37I exhibited a lower specific activity respect to the wild-type enzyme (0.03 versus 0.08 nmol/min/μg), while, for the mutant carrying the two amino acid changes (L37I-G38A), no activity was detected (Figure 2D). BLAST alignments run to identify highly conserved amino acids at the mutagenized positions showed that the mutagenized residues (L37 and G38) are highly conserved in all the GAMT proteins sequenced, with the exception of Leu37Met (L37M) in the *Coturnix japonica* (predicted sequence), *Pundamilia nyererei* (predicted sequence), and *Branchiostoma floridae* (predicted sequence). Therefore, L37M substitution was tested instead of L37I for its ability to preserve GAMT stability. Furthermore, the predicted effect of L37M substitution on protein flexibility is shown to be lower compared with L37I or L37I-G38A substitutions (Figure S2). The purified wild-type recombinant GAMT protein is also prone to precipitation, as mentioned previously. Although GAMT functions as a monomer, hydrophobic and hydrophilic interactions among monomers promote the formation of a homotetramer, as also seen in the crystallographic structure (Figure 3A). By reasoning that the precipitation of rGAMT may also stem from the formation of this ternary structure, *in silico* studies focused on targeting the main intermolecular interactions among four GAMT monomers were performed. These led to the identification of two

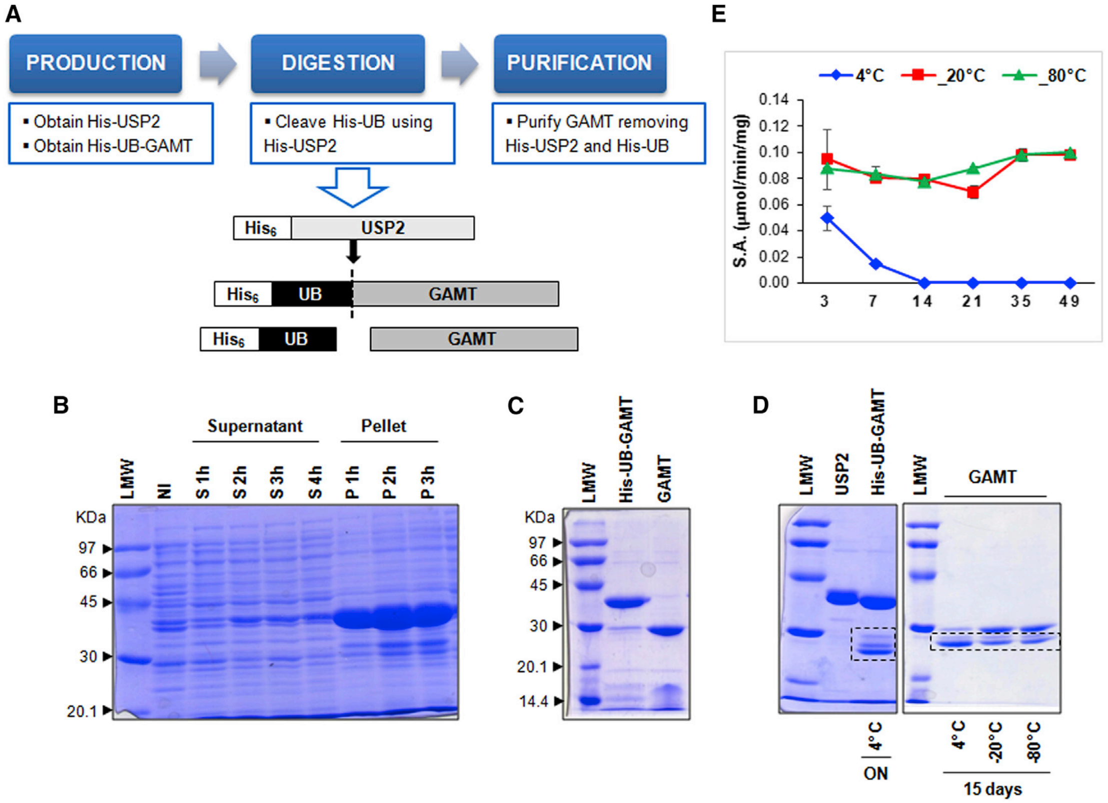

Figure 1. Expression strategy of native GAMT

(A) Expression strategy to obtain no-tag GAMT from the pET45b/His-UB-GAMT construct. (B) SDS-PAGE of a representative time course induction experiment, where supernatant (S) and pellet (P) fractions are compared. NI is the not-induced sample. (C) NI-affinity chromatography performed before and after USP2 digestion. (D) Stability assay: His-UB-GAMT and no-tag GAMT were stored at different temperatures, for the times indicated. (E) GAMT specific activity (S.A.) at different storage conditions, expressed as micromoles/minute/milligrams. Data are means ± SD of 3 independent experiments. ON, overnight. Low-molecular-weight (LMW) protein standards are indicated by arrowheads.

residues situated in the N terminus (Ala26 and Leu37) and one in the C terminus of GAMT (Val215), predicted to mediate hydrophobic interactions between two GAMT monomers (Figures 3A and S3). To further address the solubility issue, we also conducted bioinformatic analyses of hotspots of protein aggregation, resulting in the identification of an additional residue (Ile187) with a high aggregation potential (Figure 3B). The four selected residues were replaced considering the conformational free energy change (ΔΔG) caused by the single amino acid changes (calculated by Eris platform dynamic models), their influence on the intermolecular energy interactions (calculated by FoldX), and the positive contribution on solubility (calculated by Aggrescan3D). On the basis of these contributions, the best candidates were A26N, L37M, I187Q, and V215Q (Figure 3C). The predicted ΔΔG of protein folding after the planned multi-site mutagenesis is −1.93 kcal/mol. Upon mutagenesis, three candidate clones were selected for further studies:

CL32: A26N-L37M-I187Q-V215Q

CL51: L37M-I187Q-V215Q

CL76: A26N-L37I-I187Q-V215Q

Hereon, the term GAMT will refer to mutagenized forms, which are depicted in the scheme of Figure 4A. Although most of the recombinant His-UB-GAMT protein continued to aggregate in the inclusion bodies, the small-scale induction of the mutagenized constructs, performed as described in materials and methods, showed an improvement in terms of solubility for the three clones (CL32, CL51, and CL76), with an increased amount of His-UB-GAMT in the soluble fraction (Figure 4B). At the protein level, all GAMT mutants showed no degradation when maintained up to 42 h at 37°C, both in the His-UB-tagged form and after USP2-mediated His-UB removal (Figure 4C). The enzymatic activities of CL32, CL51, and CL76 evaluated at the end of purification (time 0) were 0.15, 0.16, and 0.09 U/mg protein, respectively. The enzyme assay of GAMT showed that CL32 and CL51 maintained about 60% and 56% of activity after three days at 37°C, respectively, whereas CL76 exhibited about 33% activity under the same conditions. On the basis of these results, CL32 was selected as lead candidate for the subsequent studies, from the laboratory-scale expression to the scaling-up production by synthetic media.

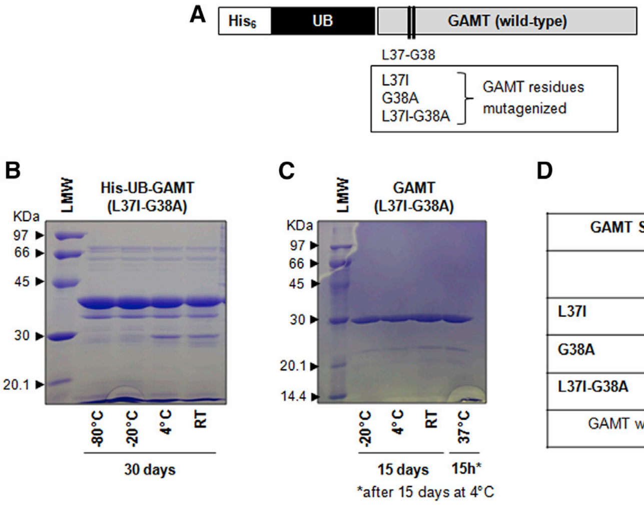

D

GAMT Specific Activity (U/mg)		
	His-UB-GAMT	NO-tag GAMT
L37I	0.03±0.003	0.03±0.003
G38A	0.03±0.003	0.03±0.003
L37I-G38A	n.d.	n.d.
GAMT wild-type S.A. 0.08 ± 0.02		

Figure 2. GAMT mutagenesis and protein stability
(A) His-UB-GAMT expression cassette: the designed amino acid substitutions within GAMT coding sequence are highlighted. (B and C) Stability assay: His-UB-GAMT and no-tag GAMT carrying the double amino acid substitutions (L37I-G38A) were stored at different temperatures, for the times indicated, and then analyzed by SDS-PAGE. Low-molecular-weight (LMW) protein standards are indicated by arrowheads. RT, room temperature. (D) Specific activity of the three mutagenized GAMT, both in the precursor and tag-less form (mean ± SD) .

Expression optimization of GAMT CL32 in BL21(DE3)

As first attempts, expression studies were performed at laboratory scale in Luria and Bertani (LB) medium using the standard conditions described in materials and methods, i.e.: 37°C incubation during both the growth and induction phase, started by 1 mM isopropyl ß-D-1-thiogalactopyranoside (IPTG) addition. The yield of His-UB-GAMT after the first Ni-affinity chromatography was around 33 mg/L of induced culture, from which about 20 mg of native GAMT were recovered after USP2 digestion (~60% recovery yield). The specific activity of the enzyme produced at laboratory-scale level, at the end of the purification step (time 0), was lower than the previous production (0.09 versus 0.15 μmol/min/mg), owing to a lower degree of purity of the product. In addition, stability studies showed that the enzyme activity decreased by about 30% after 24 h at 37°C (0.07 μmol/min/mg) and then remained mostly stable until three days, with values of 0.07 μmol/min/mg (48 h) and 0.06 μmol/min/mg (72 h), respectively. Subsequently, to move toward a product for clinical use, different scaling-up productions (using 5.5 L culture volumes) were performed in synthetic media, using a head plate layout 7-L bioreactor (Applikon Italia). The scaling-up phase in the synthetic medium allowed obtaining a good yield of protein after the two Ni-affinity chromatography (~7.0 mg of native GAMT per liter of induced culture), with specific activity values overlapping those obtained with LB medium in laboratory-scale experiments. A representative large-scale expression/purification experiment for GAMT CL32 is shown in Figures 5A and 5B. The purified GAMT, stored at −80°C, remained stable for up to 4 months; then, its activity decreased by 35% at 8 months, remaining at this level for up to 18 months; subsequently, there was a decrease of 65% and 74% after 24 and 36 months, respectively.

Basal characterization of the GAA/creatine metabolic pathway in RBCs

To engineer RBCs as bioreactors for the degradation of GAA and simultaneous production of creatine, preliminary biochemical characterizations were performed. The ability of GAA uptake by RBCs is shown in Figure 6. A time- and concentration-dependent influx of GAA into RBCs has been observed: by increasing GAA concentration in the incubation medium in the range 0–100 μM, higher amounts of GAA were recovered into the cells (Figure 6A). Notably, at GAA concentrations reproducing GAA plasma content in GAMT-deficient patients (~50 μM as higher value); 38.42 ± 6.4 nmol GAA/mL packed RBCs were found inside cells after 1 h of incubation at 37°C. GAA influx velocity into RBCs is perfectly linear with increasing GAA concentrations, at least up to 50 μM GAA, where an influx of 0.772 ± 0.1 nmol/min/mL RBCs at 100% hematocrit (Ht) was observed (Figure 6B). To carry on the study on the metabolic modeling of GAMT-loaded RBCs (see below), the cellular content of SAM was evaluated. It was 11.7 nmol/mL of packed RBCs, in agreement with Wise et al.[17] (11.86 nmol/mL packed RBCs). Likewise, erythrocyte MAT catalytic activity has been evaluated by measuring the incorporation of radioactivity from L-[methyl-^{3}H] methionine into the product S-adenosyl-[methyl-^{3}H]-methionine. In the RBC lysate fraction, the MAT specific activity was 0.27 pmol/mg total proteins, in agreement with Cheng et al.[18] (0.23 pmol/mg total protein). Human RBCs were then loaded with purified recombinant GAMT (340 μg added in the dialysis step), obtaining cells with 0.01 IU/mL cells at 100% Ht, corresponding to 0.138 mg of GAMT per milliliter of cells at 100% Ht. By increasing the amount of protein added during the hypotonic lysis and dialysis step (e.g., 660 μg) and by replacing the dialysis buffer after 45 min with a fresh one (reaching RBCs 97 mOsm), cells loaded with 0.043 IU/mL RBCs at 100% Ht, corresponding to 0.53 mg/mL RBCs, were obtained. The ability of these engineered RBCs to metabolize GAA and produce creatine has been evaluated by MS/MS analysis. The results obtained show that RBCs loaded with recombinant human GAMT were ineffective in the metabolism of extracellular GAA (data not shown). We, therefore, reasoned that the intracellular SAM content could be rapidly depleted by the GAMT-catalyzed reaction and not readily replenished by the endogenous MAT. To test whether endogenous MAT kinetics limits the entire pathway and to overcome this metabolic bottleneck, we decided to produce the recombinant MAT enzyme to be encapsulated in the erythrocytes together with the GAMT enzyme.

Cloning and expression optimization of human MAT2A

Three human isoforms of MAT are present in human tissues.[19] Of these, MAT2A is ubiquitously expressed. MAT2A forms a functional

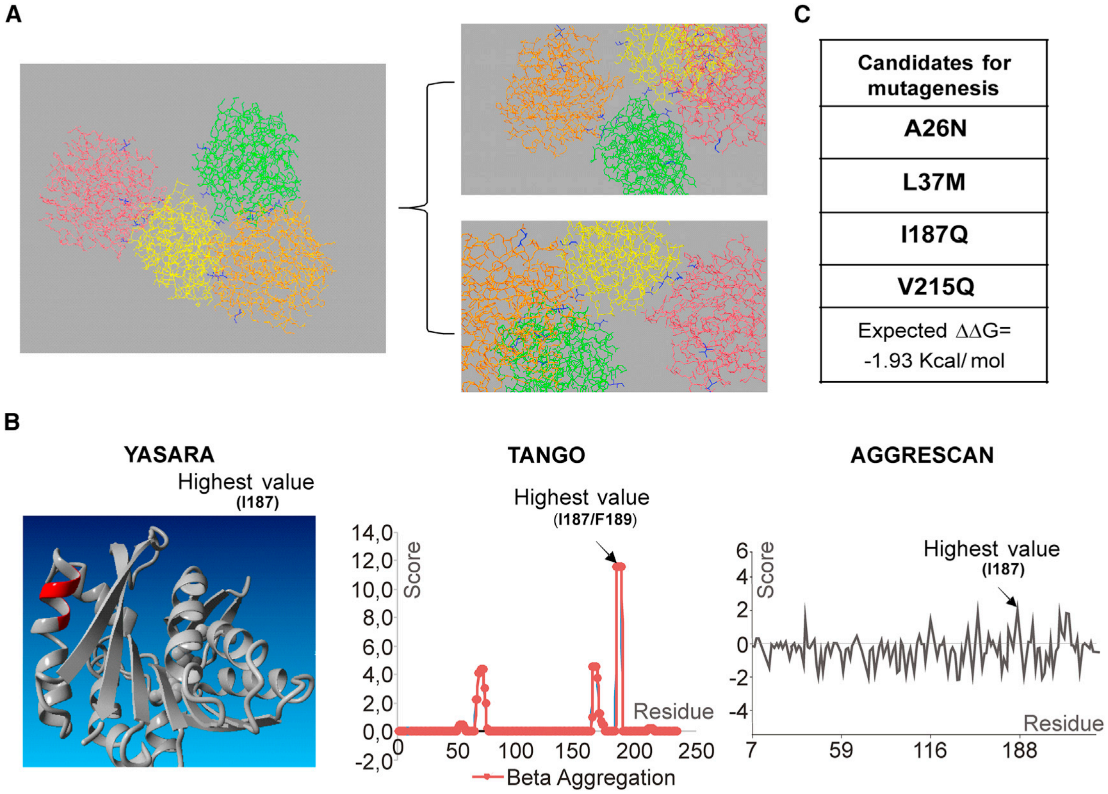

Figure 3. GAMT engineering to increase protein stability and solubility

(A) *In silico* analysis of GAMT intermolecular interactions, based on the crystallographic structure. Changed amino acids are in blue. (B) Bioinformatic solubility studies with YASARA (http://www.yasara.org/biotools.htm), TANGO (http://tango.crg.es), and Aggrescan3D (http://biocomp.chem.uw.edu.pl/A3D2/) tools. (C) Summary of the candidate residues for mutagenesis and calculation of the expected free energy change (ΔΔG).

homotetramer in its active form flanked on either side by a regulatory protein, referred to as MAT2B. MAT2B regulates the activity of MAT2A by increasing the susceptibility of MAT2A to product inhibition by SAM but does not provide significant rate enhancement. To avoid MAT inhibition by SAM, we have cloned and produced only the catalytic subunits MAT2A. Human MAT2A CDS (NCBI RefSeq DQ083239.1) was successfully cloned into the pET45b-UB, an expression vector engineered to produce the recombinant enzyme directly fused to the C terminus of the ubiquitin partner, to provide an easy purification of the authentic protein, without any tag[20] (Figure 7A). The no-tag enzyme was obtained by USP2 digestion of the recombinant His-UB-MAT precursor purified from the soluble fraction by Ni-affinity chromatography. Although most of the recombinant product was partitioned in the insoluble fraction (Figure 7B), the amount of protein in the soluble part was sufficient for our purposes. Optimization of MAT expression to improve product yield was performed directly in synthetic medium, both in laboratory-scale experiments and large-scale productions. Figures 7B and 7C shows a typical scaling-up induction and purification experiment. To optimize enzyme specific activity, in the scaling-up process, a diethylaminoethyl (DEAE) chromatography was carried out instead of the Ni-immobilized metal-ion-affinity chromatography (IMAC) to obtain the no-tag enzyme devoid of the His-tagged byproducts. The specific activity of recombinant MAT was 0.2 ± 0.028 U/mg (mean of three independent experiments).

Human RBC engineering by co-entrapment of GAMT and MAT

Human RBCs were subjected to the loading procedure with both GAMT and MAT individually and together, with defined ratios, as described in materials and methods. The amount of the entrapped proteins at the end of the procedure is shown in Figure 8A. In particular, even starting from the same milligrams of protein and units (GAMT and MAT have the same specific activity), a better entrapment of GAMT was obtained, probably due to the lower MW of GAMT (29 kDa) compared with MAT (52 kDa) and, furthermore, the fact that MAT exists as a tetramer in its native form. The ability of these engineered RBCs to metabolize L-methionine (L-Met) and $^{13}C_2$-GAA and produce $^{13}C_2$-creatine has been evaluated by MS/MS analysis of RBC suspensions taken at different incubation times at 37°C. RBCs loaded only with recombinant human GAMT or MAT were ineffective in the metabolism of extracellular GAA, while RBCs loaded with both enzymes were able to produce $^{13}C_2$-creatine starting from GAA and methionine (Figures 8B–8D). GAMT and MAT entrapped at different concentrations

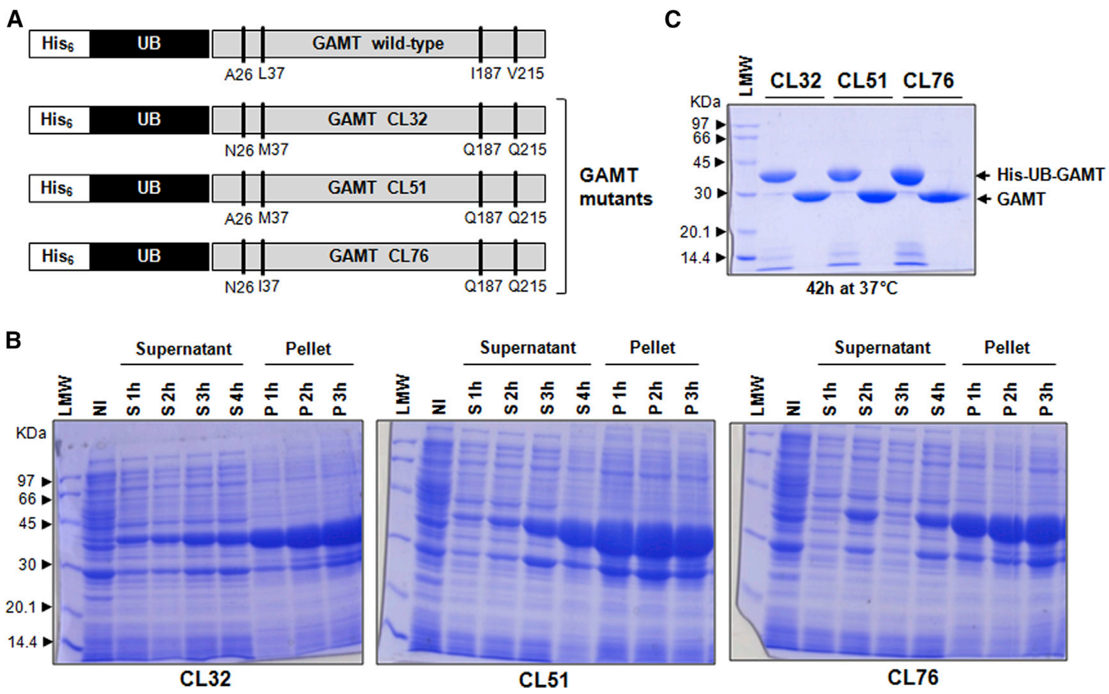

Figure 4. Mutagenesis to increase GAMT solubility and stability

(A) Schematic representation of the different His-UB-GAMT expression cassettes. At the top is the wild-type GAMT expression cassette, where the amino acid residues designed for mutagenesis are highlighted. Below are the three selected mutant clones (CL32, CL51, and CL76), with the carried amino acid substitutions. (B) SDS-PAGE of a representative time course induction experiment, for each mutagenized construct, where supernatant and pellet fractions are compared; NI is the not-induced sample. (C) Stability assay: His-UB-GAMT and no-tag GAMT carrying the amino acid substitutions indicated in (A) were stored at 37°C for 42 h. Low-molecular-weight (LMW) protein standards are indicated by arrowheads.

were capable of metabolizing from 23% to 29% of the starting $^{13}C_2$-GAA amount, after 5 h incubation at 37°C (Figure 8B). The results obtained demonstrate that RBCs loaded with GAMT and MAT in a wide range of ratios could be used to metabolize GAA. From these data, it could be concluded that the entire metabolic pathway from GAA to creatine is operative in RBCs loaded with recombinant human GAMT and MAT enzymes and that methionine physiologically present in human plasma can sustain the formation of creatine (Figures 8C and 8D). The ATP needed to support MAT activity is provided by RBC glycolysis, which relies on glucose, which is also physiologically present in human plasma. Regarding L-Met consumption, all RBC suspensions were able to metabolize it after 21 h of incubation, also due to the MAT enzyme naturally present in RBCs (not shown); however, this ability was particularly evident in the first incubation times (0–4 h) in which it was possible to appreciate the effective contribution of the entrapped MAT. This metabolic behavior is corroborated by a metabolic modeling simulation of GAMT-loaded RBCs, developed to define the limiting steps in the biotransformation of GAA. A Bio-PEPA (https://homepages.inf.ed.ac.uk/jeh/Bio-PEPA/biopepa.html)[21] model of creatine synthesis pathway, based both on experimental and literature data,[22] has been created and simulations performed on it. By assuming a constant supply of L-Met and ATP and a net influx of GAA, the model represented the behavior of a unit (10 mL) of 100% Ht RBCs loaded with 1 mg of GAMT and variable amounts of MAT, over 10 h from injection in a patient with a plasmatic concentration of GAA of 50 μM. Figure S4A shows that SAM is constantly depleted; therefore, MAT kinetics limits the entire pathway. The overall kinetics allows the RBCs to seize GAA from plasma so quickly, in fact, that it is reduced by two-thirds after 10 h and a great amount of creatine is produced. In Figure S4B, the amount of creatine produced in simulations with a fixed amount of GAMT and various amounts of recombinant MAT, with respect to the control simulation with endogenous human MAT, is shown. Both the control simulation and the one with 0.05 mg of MAT produced negligible amounts of creatine while the higher MAT concentrations allowed a quick creatine synthesis supported by initial (first 20 min) depletion of intracellular SAM, followed by a slower synthesis limited by MAT kinetics until depletion of intracellular GAA (occurring at 3 h in the simulation using 5 mg of MAT), after which GAA uptake becomes the limiting factor. As shown in the graph of Figure S4B, the activity of GAMT indeed appears to be proportional to the activity of MAT encapsulated over a wide range of quantities (0.1–1 mg of MAT protein loaded), but in the presence of the two highest amounts of MAT (2.5 and 5.0 mg), it is observed instead a kinetics of MAT that exceeds that of GAMT, causing an accumulation of SAM, until saturation is reached.

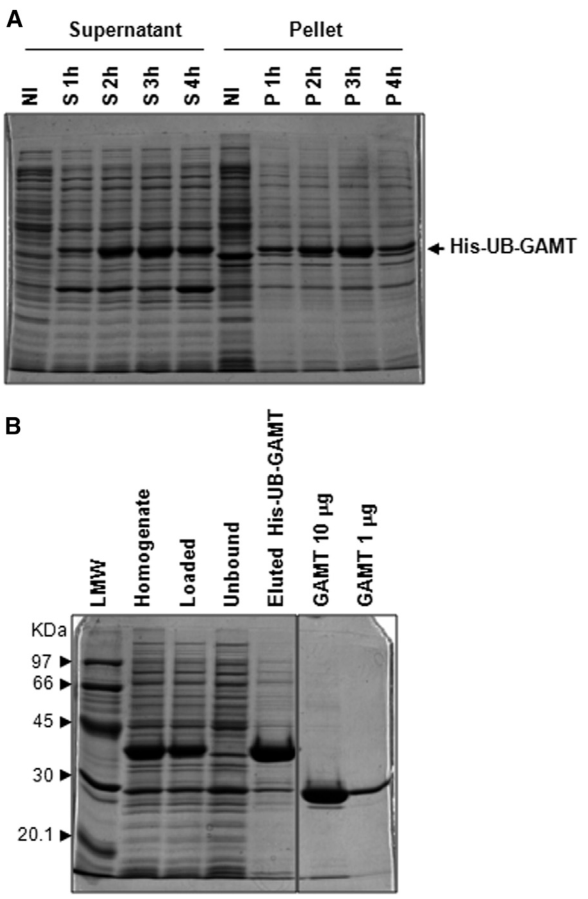

Figure 5. Scaling-up induction and purification of GAMT CL32

(A) Time course induction of the mutagenized construct CL32 (in 5.5 L LB, using the bioreactor). Supernatant and pellet fractions are resolved by SDS-PAGE; NI is the not-induced sample. (B) Purification of His-UB-GAMT CL32 by immobilized metal-ion-affinity chromatography (IMAC), with the Akta Purifier. Low-molecular-weight (LMW) protein standards are indicated by arrowheads.

DISCUSSION

Metabolic engineering was originally developed for the directed modification of metabolic pathways for the microbial synthesis of various products.[23] The same approach was later used for the production of protein therapeutics in mammalian cells.[24] Metabolic engineering of human cells is less famous than microbial engineering but could be a convenient approach to restore the limited abilities of selected cells in pathological conditions or to improve rate-limiting pathways in the same. Human erythrocytes possess useful features that permit the modulation of their metabolic properties by direct entrapment of recombinant enzymes. In fact, circulating erythrocytes do not contain a nucleus and an active transcriptional and translational machinery so that conventional and new methods that take advantage of the use of gene delivery systems or CRISPR/Cas-based technologies are unusable.[25] In contrast, human erythrocytes, under controlled conditions, can be directly overloaded with recombinant proteins maintaining all the properties of native erythrocytes, including *in vivo* survival.[26] Interestingly, this approach is already in clinics, and conclusive clinical trials (phase 3) are underway.[10] The key conditions for the potential therapeutic use of human erythrocytes loaded with recombinant enzymes are essentially three. The first requirement is the availability of an enzyme that can be encapsulated into the erythrocytes and is stable; second, the enzyme substrate usually present in the plasma should be available into the cell (i.e., the substrate should be able to cross the erythrocyte membrane by simple diffusion and/or transport-mediated processes). Furthermore, the enzyme product should not affect the enzyme-engineered erythrocytes in terms of cellular stability. Ideally, the enzyme product should diffuse out of the cell and be released into the circulation to prevent water retention in an undesirable osmotic process. Moreover, the enzyme replacement therapy via infusion of autologous erythrocytes has no potential risk of transmissible disease and HLA sensitization because patients will receive autologous engineered RBCs exclusively. The main limit of this approach is that the treatment should be repeated once a month. On the other hand, the *in vivo* gene therapy approaches developed in the last 30 years demonstrated that genetic modifications would provide effective treatments for many inherited human diseases, offering durable and possibly curative clinical benefit with a single treatment, achieved through various gene delivery vectors, mostly viral-based vectors.[27] However, the three key vector strategies, based on adenoviruses (Ads), adeno-associated viruses (AAVs), and lentiviruses, as well as being at the forefront of preclinical and clinical successes, still present many challenges that limit these approaches from attaining their full potential.[28] Regarding Ad vectors, in addition to the advantage of offering large cargo capacities for transient targeted gene delivery, immunogenicity and cellular toxicity continue to be major obstacles for Ad-based gene therapy, which needs a proper control for these approaches to be successful. The immunogenicity toward the vector remains the largest challenge also for AAV-based gene therapies; however, the growing number of clinical trials using recombinant AAVs (rAAVs) is a positive sign that these vectors represent a very promising gene delivery strategy.[29] Lentiviral vectors, the other main viral platform of gene delivery, offer the advantage of long-term transgene expression, being integrating vectors. Moreover, extensive optimization efforts have been performed to mitigate the risk of insertional mutagenesis.[30] In this respect, the infusion of erythrocytes loaded with the recombinant therapeutic protein(s) is safer, enabling stringent controls during the *in vitro* manipulation of autologous cells prior to infusion into patient.[10] Another important issue faced in designing efficient and safe viral vectors is the achievement of tissue or cell-type specificity to produce the therapeutic protein/enzyme where needed. Capsid reshape has been exploited to impact on and fine-tune tissue-cell tropism,[31] and designing tissue-specific promoters[32] is the main approach for improving on target tissue-expression. Of note, all viral vector platforms are currently being developed toward monogenic diseases, while the simultaneous delivery of two genes (such as GAMT and MAT, as herein reported) has not yet been established. Therefore, the proposed strategy, based on RBC engineering with both therapeutic enzymes necessary to correct the metabolic defect shows unprecedented advantages with respect to the classical gene delivery modality. Here we validate this strategy by considering a real case by implementing a strategy that could

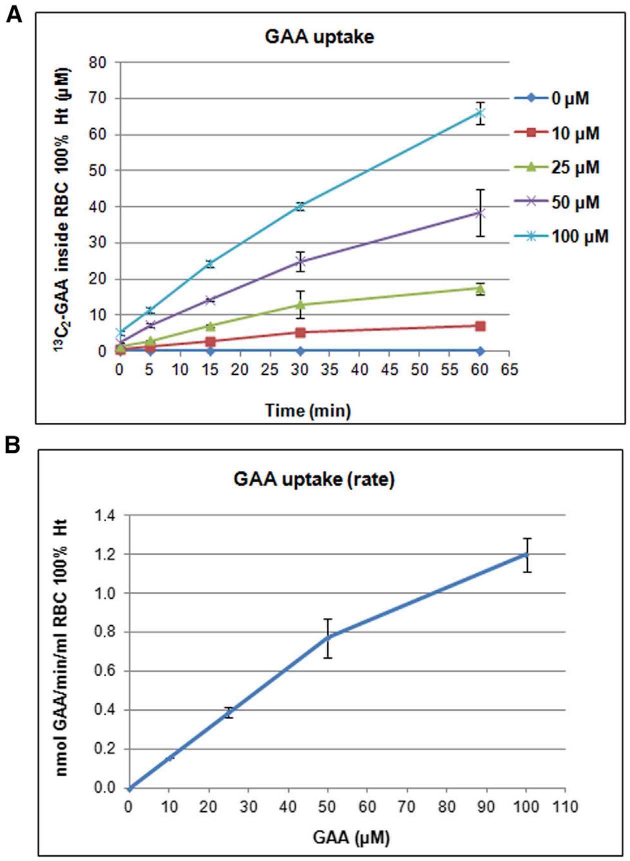

Figure 6. GAA uptake by human RBCs

(A) RBCs were incubated for 1 h at 37°C in the presence of different $^{13}C_2$-GAA amounts (0, 10, 25, 50, and 100 µM). At different incubation times, $^{13}C_2$-GAA concentration inside RBCs was evaluated by ESI-MS/MS. (B) Velocity of $^{13}C_2$-GAA uptake inside RBCs. Values are mean ± SD of 3 separate experiments.

eventually represent an innovative therapeutic approach to treat the GAMT deficiency, a rare neurometabolic disease with a severe clinical outcome. The pathogenesis of brain damage in this condition results from the cumulative effect of creatine depletion and GAA accumulation, this last being particularly toxic for the central nervous system.[6,7] At first, we developed a strategy to replace the deficient enzyme by producing a recombinant form of the native human GAMT to be encapsulated into autologous RBCs. Because the recombinant enzyme we initially produced showed low solubility and limited stability, we started a complex process of enzyme engineering to optimize these features. The selected mutant clone (CL32) represented the best choice among all the mutant forms produced; indeed, mutagenesis of four critical amino acids in the wild-type enzyme was able to increase both the solubility and the stability of the recombinant protein over time. Moreover, the mutant GAMT showed a good affinity for its substrate GAA and a good specific activity and was successfully encapsulated into RBCs. However, experimental data and a metabolic modeling of GAMT-loaded RBCs revealed that the intra-erythrocytic SAM was not able to support GAMT activity, leading to the need to encapsulate into RBCs also a recombinant form of human MAT to compensate for this deficiency. Human MAT consists of a functional homotetramer (MAT2A) flanked on either side by a regulatory protein, referred to as MAT2B. The entire complex is too large to be efficiently encapsulated into human RBCs; thus, we produced only the catalytic subunits MAT2A. Indeed, the co-entrapment of both enzymes resulted in GAMT-MAT-loaded human RBCs able to perform *in vitro* as bioreactors, capable of metabolizing GAA and producing creatine. It is noteworthy that only the presence of both enzymes entrapped into RBCs results in a fully functional cellular bioreactor, while RBCs loaded with GAMT or MAT alone were not effective in the production of creatine from GAA. We noted that all other RBC metabolic properties should be maintained to have a functional cellular bioreactor. In fact, the presence of glucose in the medium fuels the glycolytic pathway, while extracellular methionine at physiological concentrations supports the intracellular production of SAM. Moreover, previous preclinical and clinical studies[10,33] on protein-loaded RBCs confirm that engineered erythrocytes maintain a vitality, such as to be a viable therapeutic product, despite some differences existing between untreated native and loaded RBCs, which justify their slightly reduced survival in circulation but do not compromise their therapeutic efficacy.[34–36] The key physiologic mechanisms controlling the *in vivo* behavior of RBCs engineered as drug delivery system, as well as the design parameters developed to overcome the main critical steps, have been recently reviewed.[37] Therefore, we can envisage that periodic infusions of RBCs with the co-entrapped proteins could eventually represent an effective therapeutic strategy for the treatment of GAMT deficiency in patients in need. To this end, we have now planned preclinical studies to evaluate the *in vivo* efficacy of murine GAMT-MAT-loaded RBCs in a mouse model of GAMT deficiency.

Engineered RBCs could likely be used in the treatment of patients with high GAA plasma concentrations, as in GAMT deficiency, but they can also provide an additional biological source of creatine, from said engineered RBCs. Furthermore, our results also highlight the ability of GAMT-MAT-loaded RBCs to efficiently metabolize L-Met, suggesting a further perspective of the proposed model in those pathologies that could benefit from L-Met depletion in the circulation, as in some kinds of tumors, as demonstrated by others and by us.[7,38] Finally, the data provided in this paper support our previous statements: (1) for the construction of cellular bioreactors using autologous RBCs, it is essential that the initial substrate (in our case GAA) can enter into the engineered RBCs to fuel the bioreactor; (2) that the RBC is endowed with stable and easily encapsulable recombinant enzymes (eventually generated by suitable protein engineering approaches); and (3) that the product of the implemented metabolic pathway can diffuse out of the cell. Additional advantages are the possibility of removing toxic metabolites from biological fluids by converting them into useful or harmless products, GAA to creatine in the present case, or phenylalanine to trans-cinnamic acid in phenylketonuria (PKU) treated by phenylalanine ammonia lyase (PAL).[39] We are convinced that these data represent the basis for the development of a possible therapeutic agent and that the collected data provide a rationale for the design of other useful cellular bioreactors.

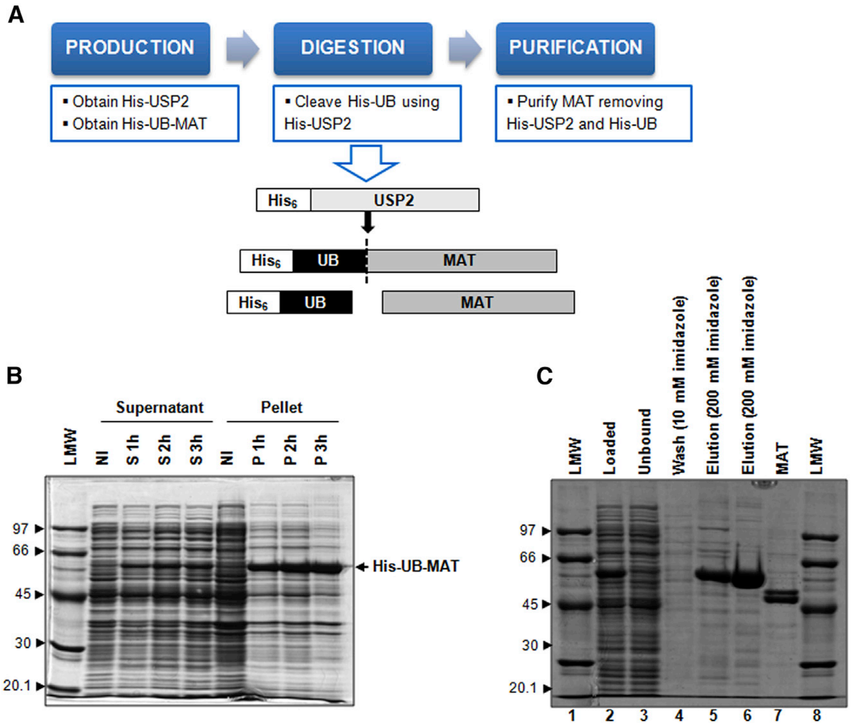

Figure 7. Expression strategy, scaling-up induction, and purification of MAT

(A) Expression strategy to obtain MAT no-tag from the pET45b/His-UB-MAT2A construct. (B) Time course induction of the pET45b/His-UB-MAT2A construct (in 5.5 L synthetic medium, using the bioreactor). Supernatant and pellet fractions are resolved by SDS-PAGE; NI is the not-induced sample. (C) Purification of His-UB-MAT by immobilized metal-ion-affinity chromatography (IMAC). The final MAT product obtained by DEAE chromatography, with the Akta Purifier, is loaded in lane 7. Low-molecular-weight (LMW) protein standards are indicated by arrowheads.

MATERIALS AND METHODS

Materials

The Change-IT Multiple Mutation Site-Directed Mutagenesis Kit was obtained from Affymetrix (USB Corporation, Cleveland, OH, USA). Mutagenic primers as well as sequencing primers were synthesized by Sigma-Aldrich (Steinheim, Germany). The QIAprep Spin Miniprep Kit and the Plasmid midi-kit used for purification of plasmid DNA were from QIAGEN (Valencia, CA, USA). Tryptone, Yeast extract, Agar, Vegetable Peptone of microbiology grade as well as ampicillin and all the chemical components of synthetic medium were from Sigma-Aldrich. IPTG was obtained from VWR International (Radnor, Pennsylvania, USA). The Ni Sepharose High-Performance resin used for IMAC was purchased from GE Healthcare Life Sciences (Amersham, UK). Centricon centrifugal filter devices, with Ultracel 10K membrane–10,000 nominal molecular weight limit (NMWL), used for recombinant enzyme concentration, were from Merck Millipore (Burlington, Massachusetts, USA). The low-molecular-weight (LMW) Calibration Kit for SDS Electrophoresis was from GE Healthcare. Sodium phosphate dibasic anhydrous (Na_2HPO_4), sodium dihydrogen phosphate (NaH_2PO_4), SDS, phosphoric acid (H_3PO_4), acetonitrile (high-performance liquid tomography [HPLC] grade), Tris-HCl, dithiothreitol (DTT), EDTA, SAM, GAA, creatine, perchloric acid (PCA), sodium acetate, trichloroacetic acid (TCA), and octanesulfonic acid sodium salt were obtained from Sigma-Aldrich. Hydrophilic polypropylene membrane filters (13 mm, 0.2 μm) were obtained from Merck Life Science (Milan, Italy). [Methyl-^{3}H] methionine and scintillation fluid Emulsifier Scintillator Plus were obtained from PerkinElmer (Boston, MA, USA). The ion-exchange columns, Discovery DSC-SCX solid-phase extraction (SPE) tubes, were purchased from Supelco Analytical (Sigma-Aldrich). All other chemicals used were obtained from Carlo Erba (Milan, Italy) and were of analytical grade.

Preparation of pET45b/His-UB-GAMT and pET45b/His-UB-MAT2A expression constructs

Total RNA was extracted from the cervical cancer cell line HeLa by RNeasy Plus Mini Kit (QIAGEN) and quantified by NanoDrop Technologies (Wilmington, DE, USA). RNA (1 μg) has been retrotranscribed using SuperScript First-Strand Synthesis System (Invitrogen, Carlsbad, CA, USA), oligo-dT (0.5 μg/reaction) and random hexamers (0.15 μg/reaction) in a final volume of 20 μL. For recombinant GAMT expression, specific degenerate primers (reported in Table S1) were designed to obtain the PCR product of interest for the human GAMT CDS (NCBI RefSeq NM_000156.5), using the highly processive and proofreading Platinum Pfx DNA Polymerase (Invitrogen), according to the manufacturing instructions. The amplified GAMT CDS was initially cloned in the expression vector pET45b downstream of the His-tag coding sequence, using the PmlI and HindIII restriction sites. Next, the GAMT coding sequence was amplified by Pfx with a new forward primer (carrying a NcoI cutting site, shown in Table S1) and the same reverse primer used above and cloned in the pET45b-UB, an expression vector engineered to contain the ubiquitin coding sequence downstream of the His-tag. The final construct was referred to as pET45b/His-UB-GAMT. This strategy allows the production of GAMT protein, deleted of the initial L-Met (ΔMet1), as occurs in the mature form of the human enzyme, directly fused at the C terminus of the $(His)_6$-UB. The PCR product, purified by the QIAquick PCR Purification Kit (QIAGEN) and quantified by NanoDrop, was digested with NcoI, blunted using Mung Bean Nuclease and then cut with HindIII before ligation with the pET45b-UB vector, linearized with Bam HI, and subjected to Klenow fill-in reaction, before digestion with HindIII and alkaline phosphatase treatment. The ligase reaction was used to transform the NovaBlue *E. coli* strain, following a standard protocol, and the transformation reaction plated on LB agar plates containing 100 μg/mL ampicillin. Positive colonies were screened by PCR using the vector-specific primers T7 promoter

A

	µmol/min/ml packed RBCs	
Sample	GAMT	MAT
GAMT 125 µg	0.0128	-
MAT 125 µg	-	0.004
GAMT 125 µg+MAT 125 µg	0.0120	0.002
GAMT 250 µg+MAT 125 µg	0.0198	0.002
GAMT 125 µg+MAT 250 µg	0.0117	0.007

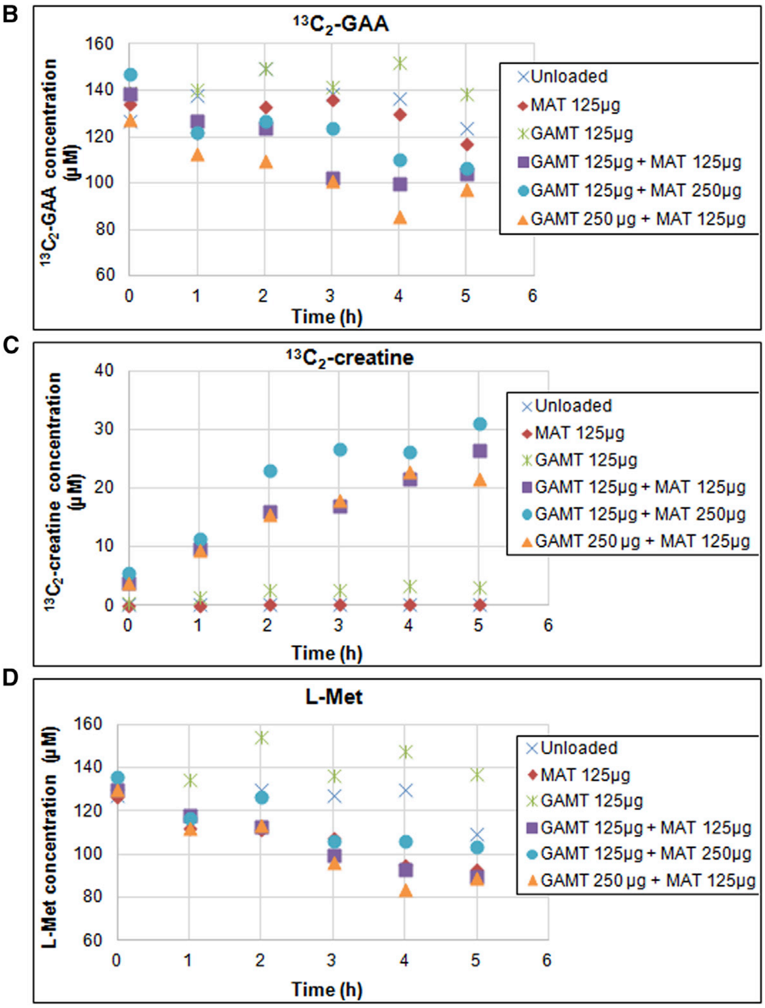

Figure 8. GAA metabolism in GAMT- and/or MAT-loaded RBCs

(A) Amounts of entrapped enzymes added in different ratios. (B) GAA consumption, (C) creatine production, and (D) L-Met consumption by loaded RBCs. GAA, creatine, and L-Met values after incubation of GAMT- and/or MAT-loaded RBC suspensions in PBS solution at 40% Ht in the presence of 200 µM $^{13}C_2$-GAA and 200 µM L-Met for 5 h at 37°C. At the planned time points, biochemical monitoring of $^{13}C_2$-GAA, $^{13}C_2$-creatine, and L-Met by MS/MS analysis of RBC suspensions were performed. In the insets, the different loading conditions are specified. The data points are representative of two different experiments that agree within 5%.

and T7 terminator. Plasmid DNA was purified from different positive clones with the QIAprep Spin Miniprep Kit (QIAGEN) and confirmed by sequencing. A positive clone was selected to be transformed into the BL21(DE3) expression host (as detailed below) to obtain the recombinant GAMT precursor, referred to as His-UB-GAMT. To generate the pET45b/His-UB-MAT2A construct, the MAT coding sequence of the human MAT2A gene (DQ083239.1) was amplified starting from cDNA obtained from HeLa cells (as above), using the Pfx DNA polymerase and specific degenerate primers designed to provide the restriction sites for SacII and NotI at the 5′ and 3′ ends, respectively. Primer sequences are reported in Table S1. The MAT2A PCR product, SacII/NotI digested, was then inserted into the pET45b-UB expression vector, downstream of the $(His)_6$-ubiquitin coding sequence. Plasmid DNA of PCR-screened-positive clones was purified with the QIAprep Spin Miniprep Kit (-QIAGEN) and confirmed by sequencing, using the primers reported in Table S1. A positive clone was chosen to obtain the expression strain.

Establishment of GAMT and MAT2A expressing BL21(DE3) *E. coli* strains and preparation of bacterial glycerol stocks for long-term storage of plasmids

BL21(DE3) competent cells (50 µL) were transformed with 1 µL of a 2 ng/µL dilution of sequence-confirmed GAMT and MAT2A expression vectors, following a standard protocol. A few clones of BL21(DE3), bearing the pET45b/His-UB-based GAMT and MAT2A expression constructs, were tested for recombinant protein expression. A single colony was picked from a freshly streaked

ampicillin containing plate. The bacterial culture was incubated at 37°C with shaking at 250 rpm, until optical density at 600 nm (OD_{600}) reached approximately 0.7–0.8 (4–6 h required). Then, 900 μL aliquots of the bacterial culture were added to glycerol (at a final concentration of 40%), previously dispensed in the cryovials. One hundred and fifty glycerol stock tubes of *E. coli* cells, both NovaBlue and BL21(DE3), transformed with the different expression constructs, were set up, immediately frozen at −80°C, and referred to as the research cell bank (RCB).

Site-directed mutagenesis of pET45b/His-UB-GAMT construct

Mutagenesis was carried out by using the Change-IT Multiple Mutation Site-Directed Mutagenesis Kit (Affymetrix), following the manufacturer's instructions, with slight modifications. All the reactions were performed using the wild-type pET45b/His-UB-GAMT expression construct as template and the mutagenic primers designed, according to the guidelines provided in the kit protocol, to introduce the targeted amino acid substitutions. Primer sequences are reported in Table S1. Briefly, a typical reaction was assembled, adding 1× Change-IT buffer, 0.25 μM GAMT mutagenic primer(s), 0.25 μM AMP forward primer (which anneals to the opposite plasmid strand relative to the mutagenic primer), 7.5 ng of plasmid DNA template, and 0.4 μL of Change-IT enzyme, in a final volume of 10 μL. The reaction was performed in the PCR 2700 Thermal Cycler. A 5 μL aliquot of the completed mutagenesis reaction has been removed to a fresh tube, added with 0.5 μL DpnI and incubated at 37°C for 2 h, before transformation in *E. coli* NovaBlue competent cells. A few clones were amplified for plasmid DNA purification performed by QIAprep Spin Miniprep Kit, according to the manufacturer's instructions. The mutated sequences were verified by automated DNA sequencing of the expression constructs in both directions, using an ABI PRISM 310 DNA sequencer (Applied Biosytems, Waltham, Massachusetts, USA) and the primers reported in Table S1.

Induction of rhGAMT and rhMAT at laboratory-scale level

The standard small-scale induction protocol is described below (any variation of induction parameters is reported in results). The starting culture was an isolated colony, picked from a freshly streaked plate and grown overnight (ON) at 37°C in 10 mL LB + ampicillin (100 μg/mL final concentration) on a benchtop orbital shaker. A sample of the ON culture was withdrawn and diluted 1:10 before reading OD_{600} in a spectrophotometer. The ON inoculum was then diluted into 100 mL LB + ampicillin in order to reach OD_{600} of about 0.1 and put in a 0.5-L flask. The culture was then incubated at 37°C with aeration and shaking at 250 rpm. Bacterial growth was monitored, at regular intervals, until culture density reached an OD_{600} of about 0.5–0.6. A 10 mL aliquot of culture was then transferred to a labeled 50 mL tube and let to grow apart at 37°C (not-induced control [NI)]. IPTG at 1 mM final concentration was added into the flask to induce protein production (induced sample [I]). At 1 h-intervals, up to 4 h, 10 mL of induced bacterial culture were withdrawn, transferred into a 15 mL tube, and put on ice. Cells were collected by centrifugation at 2,750 g for 20 min at 4°C, the supernatant was removed, and the pellet frozen at −20°C for later processing.

Preparation of bacterial extracts and SDS-PAGE analysis

Cell lysis was obtained by resuspension of the cell pellets (from both NI and I samples) in 0.05 volume of ice-cold lysis buffer (for GAMT: 20 mM Na/K phosphate buffer, pH 7.5; 15 mM β-mercaptoethanol; 15% [v/v] glycerol; and 500 mM NaCl; for MAT: 40 mM Tris-HCl; 2.2 mM KCl, 20% [v/v] glycerol; and 0.04% [v/v] Tween 20, 110 mM NaCl), followed by three cycles (30 s each) of sonication at 75 W, while keeping the sample on ice. Samples were centrifuged 10 min at 9,600 g at 4°C: the supernatant (S), corresponding to the soluble cytoplasmic fraction, was transferred into a new microcentrifuge tube, while the residual pellet was resuspended in lysis buffer and sonicated, as above; this further sample has been referred to as the pellet (P) sample. The protein concentration in both S and P samples was determined by the Bradford assay,[40] to normalize the samples for loading. For SDS-PAGE analysis, an aliquot corresponding to 20 μg of proteins was combined with an equal volume of 2× sample buffer (2% SDS, 100 mM Tris-HCl [pH 6.8], 20% glycerol, and 0.0025% w/v bromophenol blue) and boiled 1 min before resolving by 12% (w/v) polyacrylamide gel electrophoresis, in parallel with the LMW standard (GE Healthcare) as a size reference.

Immobilized metal-ion-affinity chromatography for His-UB-GAMT and His-UB-MAT purification

The recombinant His-tagged proteins in the supernatant fraction (His-UB-GAMT and His-UB-MAT) were purified by the IMAC, exploiting the interaction between chelated transition metal ions (Ni^{2+}) and side-chains of histidines (His) on proteins, essentially according to the manufacturer's instructions. Briefly, the column was equilibrated with the binding buffer, which corresponds to the lysis buffer, in which the sample is resuspended. After sample loading, washings were initially performed with the same binding buffer and then with binding buffer containing 40 mM imidazole. Elution of the His-tagged recombinant protein was obtained by binding buffer containing 350 mM imidazole.

USP2 digestion of His-UB-GAMT and His-UB-MAT and no-tag enzyme purification

Both His-UB-GAMT and His-UB-MAT, obtained upon Ni Sepharose-affinity chromatography, were digested with the recombinant His-tagged deubiquitinating enzyme USP2 (rHis-USP2) to remove the His-UB partner fused at the N terminus.[20] For optimal digestion, His-UB-GAMT or His-UB-MAT and rHis-USP2 were combined, allowing a mass ratio in the range of 200:1–100:1, and incubated at least 3 h at 37°C, in the elution buffer, suitably diluted by loading buffer in order to keep the imidazole concentration ≤50 mM. GAMT no-tag was purified from all the His-tag byproducts generated during digestion, undigested His-UB-GAMT and His-USP2, by NI-affinity chromatography. MAT no-tag was instead purified from all the His-tag byproducts by ion-exchange-DEAE chromatography. Briefly, the column was equilibrated with the MAT binding buffer, detailed above. After sample loading, washings were initially performed with the same binding buffer and then with binding buffer containing 50 mM NaCl. Elution of the no-tag protein was obtained by binding buffer containing 300 mM NaCl. The NaCl concentration was then

brought back to 110 mM. The final recovery yield for both GAMT and MAT was calculated upon the protein concentration assay with the Bradford method,[40] while the purity of the enzyme was assessed by SDS-PAGE. The purified proteins were stored at −80°C until use.

Determination of recombinant GAMT activity

The evaluation of enzyme activity was performed by a method based on the quantification of creatine, according to Alessandrì et al.,[41] with some modifications. Briefly, in a final volume of 250 μL, the reaction mixture contains 50 mM Tris-HCl (pH 7.5), 2 mM DTT, 0.25 mM SAM, 1 mM GAA, and GAMT (the assay has been standardized on a quantity of 10 μg). The incubation was carried out at 37°C for 15 min, and ,at different incubation times (0, 5, 10, and 15 min), the reaction was stopped by the addition of 250 μL of 1 N PCA. The reaction mixture was then centrifuged at 18,500 g for 15 min at 4°C, and 50 μL of the clear supernatant was injected into the HPLC after filtration with 0.2 μm filter into special vials. Blanks, containing the reaction mixture without enzyme, were analyzed as well. The analysis was performed by reverse-phase chromatography at 30°C using the following gradient elution system: 2 min at 100% buffer A, up to 100% buffer B over 18 min, and hold for 2 min; the gradient was returned to 100% buffer A over 3 min and the initial conditions restored in 5 min. The flow rate was 1 mL/min and the detection wavelength was 210 nm. The mobile phase consisted of buffer A: Na_2HPO_4 (7.1g), SDS (2g), and distilled water (750 mL); and buffer B: Na_2HPO_4 (7.1g), SDS (2g), distilled water (500 mL), and acetonitrile (250 mL). Both buffers reached a final pH 3 by H_3PO_4. Before use, the buffers were filtered by Polypro filters. The increase of creatine levels has been observed after different analysis times (0–15 min). Values are expressed as micromoles of creatine/minute/milligrams of protein.

Determination of recombinant MAT activity

The evaluation of enzyme activity was performed by a method based on the quantification of S-(5′-adenosyl)-L-methionine chloride dihydrochloride (SAM), according to Shiozaki et al.,[42] with some modifications. Briefly, in a final volume of 250 μL, the reaction mixture contained 100 mM Tris-HCl (pH 7.5), 50 mM $MgCl_2$, 100 mM KCl, 8 mM GSH, 20 mM ATP, 20 mM L-Met, and MAT (the assay has been standardized on amounts of 10 and 20 μg). The incubation was carried out at 37°C for 10 min: at different incubation times (0, 1, 2.5, 5, and 10 min) the reaction was stopped by the addition of 250 μL of 2 N PCA. After filtration with 0.2 μm filter, 50 μL of reaction mixture were injected into the HPLC. Blanks, containing the reaction mixture without enzyme, were analyzed as well. The analysis was performed as reported by Wang et al.,[43] with some modifications: the method was a reverse-phase chromatography, and the detection was monitored at 254 nm, at 25°C, using a gradient elution system at a flow rate of 1 mL/min and a wavelength of 254 nm. The elution conditions were as follows: 8 min at 80% buffer A and 20% buffer B, up to 60% buffer A and 40% buffer B over 0.5 min, and hold for 12.5 min. The gradient was returned to 80% buffer A and 20% buffer B over 0.5 min and the initial conditions restored in 10 min. The mobile phase consisted of buffer A (8 mM $C_8H_{17}O_3SNa$ and 50 mM NaH_2PO_4) and buffer B (CH_3OH 100%). Buffer A reached a final pH 3 by H_3PO_4 and it was filtered with Polypro filters before use. Before and after sample analysis, the column was equilibrated and standard solution of 0.05 mM of SAM in 1 N $HClO_4$ was analyzed. SAM peak was identified according to its retention time and co-chromatography with SAM standard. The increase of SAM levels after different analysis times (0–10 min) has been observed. Quantification was based on integration of peak areas, compared with the standard calibration curves of SAM.

GAA uptake by RBCs

The ability of GAA to enter into RBCs has been evaluated. In detail, RBCs at 7.5% Ht were incubated for 1 h at 37°C in the presence of different $^{13}C_2$-GAA amounts (0–100 μM). At different incubation times (0, 5, 10, 15, 30, and 60 min), 600 μL aliquots were stratified onto 600 μL 1-bromododecane and centrifuged 3 min at 7,840 g to obtain a better separation between RBCs and $^{13}C_2$-GAA. Supernatants were discarded and RBCs recovered in a physiological saline solution containing 10 mM 2-[4-(2-hydroxyethyl)piperazin-1-yl]-ethanesulfonic acid (HEPES, pH 7.4), 154 mM NaCl, and 5 mM glucose (HEPES solution), to a final 50% Ht. A 50 μL aliquot of each suspension was collected on grade 903 filter paper (Eastern Business Forms, Greenville, USA), and the obtained dried blood spots (DBSs) were submitted to $^{13}C_2$-GAA analysis by electrospray ionization tandem mass spectrometry (ESI-MS/MS), as described by Carducci et al.[44]

Evaluation of RBC SAM levels

SAM was extracted as reported by Wise et al.[17] Briefly, frozen packed RBCs were added to an equal volume of ice-cold 0.1 M sodium acetate, pH 6, and vortex-mixed. Proteins were precipitated by the addition of 40% (w/v) TCA (equal to one-fifth of the original volume of the RBC solution) followed by 30 min incubation on ice to complete precipitation. To remove precipitated proteins, the tubes were centrifuged at 25,000 g for 10 min at 4°C. The supernatant containing SAM was added with an equal volume of ice-cold peroxide-free diethyl ether to extract lipids (ether extraction step). The tubes were vortex-mixed for 20 s and centrifuged to separate the phases. The top layer was drawn off and discarded. The ether extraction step was repeated once. The samples were filtered and injected into an HPLC system. The assay was performed as reported by Wang et al.[43]: briefly, the mobile phase consisted of two solvents: solvent A (8 mM octanesulfonic acid sodium salt and 50 mM NaH_2PO_4 adjusted to pH 3.0 with H_3PO_4) and solvent B (100% methanol). The HPLC column was equilibrated with 80% solvent A and 20% solvent B. The sample was injected and separation was obtained using a step gradient as follows: 8 min at the equilibration conditions, 30 s to increase solvent B to 40%, 12.5 min at the new condition, and 30 s to return to the equilibration conditions and a minimum of 10 min before the subsequent injection. The flow rate was 1 mL/min, and detection was monitored at 254 nm. The SAM standard was dissolved in water at a concentration of 1 mM and then diluted to 0.05 mM with 0.4 M PCA.

Determination of RBC MAT activity

For the preparation of lysate, RBCs were washed three times in 10 volumes of 5 mM sodium phosphate and 150 mM NaCl, pH 7.4, and centrifuged at 1,800 g for 10 min at 4°C; the supernatant was aspirated while the RBC pellet was lysed in 4 volumes of ice-cold deionized water and incubated for 20 min on ice. The membrane fraction was pelleted by centrifugation at 20,000 g for 1 h. The cytosolic fraction, recovered from the supernatant, was dialyzed ON against 30 mM KCl, 40 mM HEPES, pH 7.4, at 4°C, before the MAT catalytic activity assay. MAT activity was assayed by measuring the rate of formation of ^{3}H-SAM from ATP and L-[methyl-^{3}H] methionine, as reported by Cheng et al.,[18] with some modifications. Briefly, the reaction mixture (total volume of 0.1 mL, pH 7.4) contained 50 μL of cell lysate and final concentrations of 30 mM $MgCl_2$, 26 mM KCl, 35 mM HEPES, 10 mM ATP, and 20 μM L-[methyl-^{3}H]-methionine (12,600 cpm/nmol). The samples were incubated at 37°C for 60 min, and the reaction was stopped by the addition of 1.0 mL ice-cold 1.6 mM citric acid in 50% ethanol. The time 0 of reaction was used as blank. After adsorption to an ion-exchange resin (column in the NH_4^+ form), S-adenosyl-[methyl-^{3}H]-methionine was eluted with 1.0 mL of 3.0 M NH_4OH into a vial containing 5 mL of scintillation fluid. The samples were counted in a Packard scintillation counter. The results of MAT activity were expressed as picomoles of SAM/minute/milligrams of protein. Protein concentration was estimated by the Lowry procedure.[45]

Loading of GAMT into human RBCs

Whole blood (WB) was obtained from healthy volunteers included in the Italian blood donor registry (A.V.I.S., Associazione Volontari Italiani Sangue) who signed an informed consent form. Blood was collected in heparinized tubes and provided by the Santa Maria della Misericordia Hospital in Urbino. GAMT was loaded into human RBCs by means of hypotonic lysis and dialysis, isotonic resealing, and re-annealing, according to Magnani et al.,[46] with some modifications: (1) dialysis procedure was carried out with the addition of 220 μL of protein solution (corresponding to 340 μg GAMT in a 1.56 mg/mL solution) to RBCs suspended in HEPES solution at 70% Ht, in a final volume of 1 mL; (2) suspension was dialyzed for 90 min at 4°C; and (4) resealing and re-annealing steps were carried out by incubating the pooled dialyzed RBC suspensions in PIGPA solution 25 min at 37°C. Final packed loaded RBCs were resuspended in HEPES solution, pH 7.4, at about 40% Ht and the amount of entrapped GAMT evaluated on RBC lysates, as detailed above.

Co-entrapment of GAMT and MAT into human RBCs

GAMT/MAT co-entrapment into human RBCs was performed as above, with the modifications stated below. The dialysis procedure was carried out with different concentrations of proteins added to RBCs suspended in Hepes solution at 60% Ht, as follows

A. 125 μg (0.025 IU) of GAMT enzyme (50 μL protein solution [2.5 mg/mL] with S.A. 0.2 IU/mg)
B. 125 μg (0.025 IU) of MAT enzyme (125 μL protein solution [1 mg/mL] with S.A. 0.2 IU/mg)
C. 125 μg (0.025 IU) of GAMT enzyme (50 μL protein solution [2.5 mg/mL] with S.A. 0.2 IU/mg) and 125 μg (0.025 IU) of MAT enzyme (125 μL protein solution [1 mg/mL] with S.A. 0.2 IU/mg)
D. 125 μg (0.025 IU) of GAMT enzyme (50 μL protein solution [2.5 mg/mL] with S.A. 0.2 IU/mg) and 250 μg (0.050 IU) of MAT enzyme (250 μL protein solution [1 mg/mL] with S.A. 0.2 IU/mg)
E. 250 μg (0.050 IU) of GAMT enzyme (100 μL protein solution [2.5 mg/mL] with S.A. 0.2 IU/mg) and 125 μg (0.025 IU) of MAT enzyme (125 μL protein solution [1 mg/mL] with S.A. 0.2 IU/mg)

All the conditions (in 1 mL final volume) were dialyzed for 90 min at 4°C, replacing the hypotonic dialysis buffer with fresh one after 45 min. After dialysis, the cells reached about 90 ± 4 mOsm. After the resealing, re-annealing, and washing phases, final packed loaded RBCs were resuspended in PBS, pH 7.4, at about 40% Ht in presence of 200 μM $^{13}C_2$-GAA and 200 μM L-Met, and incubation of these suspensions (A–E) were performed for 5 h at 37°C. At the planned time points (0, 1, 2, 3, 4, and 5 h), a 50 μL aliquot of each suspension was collected on grade 903 filter paper, and the obtained DBSs were submitted to biochemical monitoring of $^{13}C_2$-GAA, $^{13}C_2$-creatine, and L-Met by MS/MS.[44]

SUPPLEMENTAL INFORMATION

Supplemental information can be found online at https://doi.org/10.1016/j.omtm.2022.02.007.

ACKNOWLEDGMENTS

This research was partially supported by EryDel SpA. The authors also like to thank Luca Salvatore Lorello, a former student at the University of Urbino, for developing a Bio-PEPA (https://homepages.inf.ed.ac.uk/jeh/Bio-PEPA/biopepa.html) metabolic modeling simulation of GAMT-loaded RBCs.

AUTHOR CONTRIBUTIONS

M.B., L.R., and M.M. conceived the study and designed the research project; M.B., F.P., P.D.A., M.P.A., and E.D.C. performed the experiments and analyzed the data; M.M. contributed reagents/materials; M.B. and L.R. wrote the manuscript; all authors took part in discussions and revised and approved the final manuscript.

DECLARATION OF INTERESTS

Mauro Magnani and Luigia Rossi hold shares in EryDel SpA, a company with interests in the technology of RBC-based drug delivery. The other authors declare that the research was conducted in the absence of any commercial or financial relationships that could be construed as a potential conflict of interest.

REFERENCES

1. Stöckler, S., Holzbach, U., Hanefeld, F., Marquardt, I., Helms, G., Requart, M., Hänicke, W., and Frahm, J. (1994). Creatine deficiency in the brain: a new, treatable inborn error of metabolism. Pediatr. Res. *36*, 409–413. https://doi.org/10.1203/00006450-199409000-00023.

2. Mercimek-Mahmutoglu, S., Stoeckler-Ipsiroglu, S., Adami, A., Appleton, R., Araujo, H.C., Duran, M., Ensenauer, R., Fernandez Alvarez, E., Garcia, P., Grolik, C., et al. (2006). GAMT deficiency: features, treatment, and outcome in an inborn error of creatine synthesis. Neurology *67*, 480–484. https://doi.org/10.1212/01.wnl.0000234852.43688.bf.

3. Mercimek-Mahmutoglu, S., and Salomons, G.S. (2009). CRatine deficiency syndromes. In GeneReviews®, M.P. Adam, H.H. Ardinger, R.A. Pagon, S.E. Wallace, L.J.H. Bean, H.C. Mefford, K. Stephens, A. Amemiya, and N. Ledbetter, eds. (University of Washington).

4. Khaikin, Y., Sidky, S., Abdenur, J., Anastasi, A., Ballhausen, D., Buoni, S., Chan, A., Cheillan, D., Dorison, N., Goldenberg, A., et al. (2018). Treatment outcome of twenty-two patients with guanidinoacetate methyltransferase deficiency: an international retrospective cohort study. Eur. J. Paediatr. Neurol. *22*, 369–379. https://doi.org/10.1016/j.ejpn.2018.02.007.

5. Mercimek-Mahmutoglu, S., Ndika, J., Kanhai, W., de Villemeur, T.B., Cheillan, D., Christensen, E., Dorison, N., Hannig, V., Hendriks, Y., Hofstede, F.C., et al. (2014). Thirteen new patients with guanidinoacetate methyltransferase deficiency and functional characterization of nineteen novel missense variants in the GAMT gene. Hum. Mutat. *35*, 462–469. https://doi.org/10.1002/humu.22511.

6. Leuzzi, V., Mastrangelo, M., Battini, R., and Cioni, G. (2013). Inborn errors of creatine metabolism and epilepsy: epilepsy in creatine disorders. Epilepsia *54*, 217–227. https://doi.org/10.1111/epi.12020.

7. Rossi, L., Nardecchia, F., Pierigè, F., Ventura, R., Carducci, C., Leuzzi, V., Magnani, M., Cabib, S., and Pascucci, T. (2021). Intellectual disability and brain creatine deficit: phenotyping of the genetic mouse model for GAMT deficiency. Genes (Basel) *12*, 1201–1215. https://doi.org/10.3390/genes12081201.

8. Schulze, A., Ebinger, F., Rating, D., and Mayatepek, E. (2001). Improving treatment of guanidinoacetate methyltransferase deficiency: reduction of guanidinoacetic acid in body fluids by arginine restriction and ornithine supplementation. Mol. Genet. Metab. *74*, 413–419. https://doi.org/10.1006/mgme.2001.3257.

9. Schulze, A., Hoffmann, G.F., Bachert, P., Kirsch, S., Salomons, G.S., Verhoeven, N.M., and Mayatepekm, E. (2006). Presymptomatic treatment of neonatal guanidinoacetate methyltransferase deficiency. Neurology *67*, 719–721. https://doi.org/10.1212/01.wnl.0000230152.25203.01.

10. Rossi, L., Pierigè, F., Aliano, M.P., and Magnani, M. (2020). Ongoing developments and clinical progress in drug-loaded red blood cells technologies. BioDrugs *34*, 265–272. https://doi.org/10.1007/s40259-020-00415-0.

11. Syllm-Rapoport, I., Daniel, A., and Rapoport, S. (1980). Creatine transport into red blood cells. Acta Biol. Med. Ger. *39*, 771–779.

12. Qasim, N., and Mahmood, R. (2015). Diminution of oxidative damage to human erythrocytes and lymphocytes by creatine: possible role of creatine in blood. PLoS One *10*, e0141975. https://doi.org/10.1371/journal.pone.0141975.

13. Protasov, E.S., Borsakova, D.V., Alexandrovich, Y.G., Korotkov, A.V., Kosenko, E.A., Butylin, A.A., Ataullakhanov, F.I., and Sinauridze, E.I. (2019). Erythrocytes as bioreactors to decrease excess ammonium concentration in blood. Sci. Rep. *9*, 1455. https://doi.org/10.1038/s41598-018-37828-5.

14. Iqbal, F. (2015). Human guanidinoacetate n-methyl transferase (GAMT) deficiency: a treatable inborn error of metabolism. Pak. J. Pharm. Sci. *28*, 2207–2211.

15. Cellarier, E., Durando, X., Vasson, M.P., Farges, M.C., Demiden, A., Maurizis, J.C., Madelmont, J.C., and Chollet, P. (2003). Methionine dependency and cancer treatment. Cancer Treat. Rev. *29*, 489–499. https://doi.org/10.1016/s0305-7372(03)00118-x.

16. Komoto, J., Huang, Y., Takata, Y., Yamada, T., Konishi, K., Ogawa, H., Gomi, T., Fujioka, M., and Takusagawa, F. (2002). Crystal structure of guanidinoacetate methyltransferase from rat liver: a model structure of protein arginine methyltransferase. J. Mol. Biol. *320*, 223–235. https://doi.org/10.1016/S0022-2836(02)00448-5.

17. Wise, C.K., Cooney, C.A., Ali, S.F., and Poirier, L.A. (1997). Measuring S-adenosylmethionine in whole blood, red blood cells and cultured cells using a fast preparation method and high-performance liquid chromatography. J. Chromatogr. B Biomed. Sci. Appl. *696*, 145–152. https://doi.org/10.1016/s0378-4347(97)00213-2.

18. Cheng, H., Gomes-Trolin, C., Aquilonius, S.M., Steinberg, A., Löfberg, C., Ekblom, J., and Oreland, L. (1997). Levels of L-methionine S-adenosyltransferase activity in erythrocytes and concentrations of S-adenosylmethionine and S-adenosylhomocysteine in whole blood of patients with Parkinson's disease. Exp. Neurol. *145*, 580–585. https://doi.org/10.1006/exnr.1997.6466.

19. Shafqat, N., Muniz, J.R., Pilka, E.S., Papagrigoriou, E., von Delft, F., Oppermann, U., and Yue, W.W. (2013). Insight into S-adenosylmethionine biosynthesis from the crystal structures of the human methionine adenosyltransferase catalytic and regulatory subunits. Biochem. J. *452*, 27–36. https://doi.org/10.1042/BJ20121580.

20. Catanzariti, A.M., Soboleva, T.A., Jans, D.A., Board, P.G., and Baker, R.T. (2004). An efficient system for high-level expression and easy purification of authentic recombinant proteins. Protein Sci. *13*, 1331–1339. https://doi.org/10.1110/ps.04618904.

21. Ciochetta, F., Degasperi, A., Hillston, J., and Calder, M. (2009). Some investigations concerning the CTMC and the ODE model derived from Bio-PEPA. Electron. Notes Theor. Comput. Sci. *229*, 145–163.

22. Oden, K.L., and Clarke, S. (1983). S-adenosyl-L-methionine synthetase from human erythrocytes: role in the regulation of cellular S-adenosylmethionine levels. Biochemistry *22*, 2978–2986. https://doi.org/10.1021/bi00281a030.

23. Stephanopoulos, G. (2012). Synthetic biology and metabolic engineering. ACS Synth. Biol. *1*, 514–525. https://doi.org/10.1021/sb300094q.

24. Le, H., Vishwanathan, N., Kantardjieff, A., Doo, I., Srienc, M., Zheng, X., Somia, N., and Hu, W.S. (2013). Dynamic gene expression for metabolic engineering of mammalian cells in culture. Metab. Eng. *20*, 212–220. https://doi.org/10.1016/j.ymben.2013.09.004.

25. Nielsen, J., and Keasling, J.D. (2016). Engineering cellular metabolism. Cell *164*, 1185–1197. https://doi.org/10.1016/j.cell.2016.02.004.

26. Pierigè, F., Bigini, N., Rossi, L., and Magnani, M. (2017). Reengineering red blood cells for cellular therapeutics and diagnostics. Wiley Interdiscip. Rev. Nanomed. Nanobiotechnol. *9*, 1–17. https://doi.org/10.1002/wnan.1454.

27. Bulcha, J.T., Wang, Y., Ma, H., Tai, P.W.L., and Gao, G. (2021). Viral vector platforms within the gene therapy landscape. Signal Transduct. Target. Ther. *6*, 53. https://doi.org/10.1038/s41392-021-00487-6.

28. Arjmand, B., Larijani, B., Sheikh Hosseini, M., Payab, M., Gilany, K., Goodarzi, P., Parhizkar Roudsari, P., Amanollahi Baharvand, M., and Hoseini Mohammadi, N.S. (2020). The horizon of gene therapy in modern medicine: advances and challenges. Adv. Exp. Med. Biol. *1247*, 33–64. https://doi.org/10.1007/5584_2019_463.

29. Mendell, J.R., Al-Zaidy, S.A., Rodino-Klapac, L.R., Goodspeed, K., Gray, S.J., Kay, C.N., Boye, S.L., Boye, S.E., George, L.A., Salabarria, S., et al. (2021). Current clinical applications of in vivo gene therapy with AAVs. Mol. Ther. *29*, 464–488. https://doi.org/10.1016/j.ymthe.2020.12.007.

30. David, R.M., and Doherty, A.T. (2017). Viral vectors: the road to reducing genotoxicity. Toxicol. Sci. *155*, 315–325. https://doi.org/10.1093/toxsci/kfw220.

31. Mietzsch, M., Broecker, F., Reinhardt, A., Seeberger, P.H., and Heilbronn, R. (2014). Differential adeno-associated virus serotype-specific interaction patterns with synthetic heparins and other glycans. J. Virol. *88*, 2991–3003. https://doi.org/10.1128/JVI.03371-13.

32. Salva, M.Z., Himeda, C.L., Tai, P.W., Nishiuchi, E., Gregorevic, P., Allen, J.M., Finn, E.E., Nguyen, Q.G., Blankinship, M.J., Meuse, L., et al. (2007). Design of tissue-specific regulatory cassettes for high-level rAAV-mediated expression in skeletal and cardiac muscle. Mol. Ther. *15*, 320–329. https://doi.org/10.1038/sj.mt.6300027.

33. Rossi, L., Pierigè, F., Bregalda, A., and Magnani, M. (2021). Preclinical developments of enzyme-loaded red blood cells. Expert Opin. Drug Deliv. *18*, 43–54. https://doi.org/10.1080/17425247.2020.1822320.

34. Bax, B.E., Bain, M.D., Talbot, P.J., Parker-Williams, E.J., and Chalmers, R.A. (1999). Survival of human carrier erythrocytes in vivo. Clin. Sci. (Lond) *96*, 171–178.

35. Bourgeaux, V., Aufradet, E., Campion, Y., De Souza, G., Horand, F., Bessaad, A., Chevrier, A.M., Canet-Soulas, E., Godfrin, Y., and Martin, C. (2012). Efficacy of homologous inositol hexaphosphate-loaded red blood cells in sickle transgenic mice. Br. J. Haematol. *157*, 357–369. https://doi.org/10.1111/j.1365-2141.2012.09077.x.

36. Glassman, P.M., Hood, E.D., Ferguson, L.T., Zhao, Z., Siegel, D.L., Mitragotri, S., Brenner, J.S., and Muzykantov, V.R. (2021). Red blood cells: the metamorphosis of a neglected carrier into the natural mothership for artificial nanocarriers. Adv. Drug Deliv. Rev. *178*, 113992. https://doi.org/10.1016/j.addr.2021.113992.

37. Glassman, P.M., and Muzykantov, V.R. (2019). Pharmacokinetic and pharmacodynamic properties of drug delivery systems. J. Pharmacol. Exp. Ther. *370*, 570–580. https://doi.org/10.1124/jpet.119.257113.

38. Machover, D., Rossi, L., Hamelin, J., Desterke, C., Goldschmidt, E., Chadefaux-Vekemans, B., Bonnarme, P., Briozzo, P., Kopečný, D., Pierigè, F., et al. (2019). Effects in cancer cells of the recombinant L-methionine Gamma-lyase from Brevibacterium aurantiacum. Encapsulation in human erythrocytes for sustained L-methionine elimination. J. Pharmacol. Exp. Ther. *369*, 489–502. https://doi.org/10.1124/jpet.119.256537.

39. Rossi, L., Pierigè, F., Carducci, C., Gabucci, C., Pascucci, T., Canonico, B., Bell, S.M., Fitzpatrick, P.A., Leuzzi, V., and Magnani, M. (2014). Erythrocyte-mediated delivery of phenylalanine ammonia lyase for the treatment of phenylketonuria in BTBR-Pah(enu2) mice. J. Control. Release *194*, 37–44. https://doi.org/10.1016/j.jconrel.2014.08.012.

40. Bradford, M.M. (1976). A rapid and sensitive method for the quantitation of miCRogram quantities of protein utilizing the principle of protein-dye binding. Anal. Biochem. *72*, 248–254. https://doi.org/10.1006/abio.1976.9999.

41. Alessandrì, M.G., Celati, L., Battini, R., Baldinotti, F., Item, C., Leuzzi, V., and Cioni, G. (2004). HPLC assay for guanidinoacetate methyltransferase. Anal. Biochem. *331*, 189–191. https://doi.org/10.1016/j.ab.2004.05.012.

42. Shiozaki, S., Shimizu, C., and Yamada, H. (1984). Unusual intracellular accumulation of S-Adenosyl-L-methionine by microorganisms. Agric. Biol. Chem. *48*, 2293–2300.

43. Wang, W., Kramer, P.M., Yang, S., Pereira, M.A., and Tao, L. (2001). Reversed-phase high-performance liquid chromatography procedure for the simultaneous determination of S-adenosyl-L-methionine and S-adenosyl-L-homocysteine in mouse liver and the effect of methionine on their concentrations. J. Chromatogr. B Biomed. Sci. Appl. *762*, 59–65. https://doi.org/10.1016/s0378-4347(01)00341-3.

44. Carducci, C., Santagata, S., Leuzzi, V., Carducci, C., Artiola, C., Giovanniello, T., Battini, R., and Antonozzi, I. (2006). Quantitative determination of guanidinoacetate and creatine in dried blood spot by flow injection analysis-electrospray tandem mass spectrometry. Clin. Chim. Acta *364*, 180–187. https://doi.org/10.1016/j.cca.2005.06.016.

45. Lowry, O.H., Rosebrough, N.J., Farr, A.L., and Randall, R.J. (1951). Protein measurement with the Folin phenol reagent. J. Biol. Chem. *193*, 265–275.

46. Magnani, M., Rossi, L., Bianchi, M., Fornaini, G., Benatti, U., Guida, L., Zocchi, E., and De Flora, A. (1988). Improved metabolic properties of hexokinase-overloaded human erythrocytes. Biochim. Biophys. Acta *972*, 1–8. https://doi.org/10.1016/0167-4889(88)90095-x.

OMTM, Volume *25*

Supplemental information

Engineering new metabolic pathways in isolated cells for the degradation of guanidinoacetic acid and simultaneous production of creatine

Marzia Bianchi, Luigia Rossi, Francesca Pierigè, Pietro De Angeli, Mattia Paolo Aliano, Claudia Carducci, Emanuele Di Carlo, Tiziana Pascucci, Francesca Nardecchia, Vincenzo Leuzzi, and Mauro Magnani

Supplemental Information

Cloning strategies to optimize recombinant human GAMT expression

In a first attempt, the GAMT coding sequence was cloned in a pET45b expression plasmid directly fused to the His-tag coding sequence. The expression vector has been transformed in the BL21(DE3) *E. coli* strain and the clone showed a good expression of the N-terminal His-tagged recombinant protein, although the most part was in the insoluble fraction (Figure S1A). Different induction protocols were tested, changing the temperature and/or the length of induction, the cell density at which GAMT expression was induced, but in no case any improvement was observed (not shown). To overcome the drawbacks of the His-tag fusion strategy and to finalize the expression for therapeutic use, we designed and prepared two further constructs, using both pET45b and pET22b expression vectors in which the GAMT insert was cloned in such a way to produce the amino acid sequence of GAMT without any extra part (NO-tag GAMT). Unfortunately, the GAMT protein was expressed in a very low amount (or not expressed at all) (Figure S1B-C) and this result was not overcome by using different experimental conditions (different expression host, use of synthetic media). Moreover, a low growth rate was observed upon IPTG induction, which opened the hypothesis that GAMT might be toxic for bacteria, considering that it uses as substrate SAM that is very important in different endogenous pathways, like bacterial cell division (Newman et al., 1998). Following the hypothesis of GAMT toxicity for the host expressing cells, a construct referred to as pET22b-pelB-GAMT was generated, to target GAMT into the periplasmic space. The induction tests revealed that GAMT was expressed in the insoluble fraction as inclusion bodies (Figure S1D). Unfortunately, protein purification from the insoluble fraction and refolding requires too much time, difficult approaches and, moreover, the strategy used does not ensure that the pelB signal is completely removed. Then, a different expression strategy was attempted by cloning the GAMT CDS in the pET22b backbone in frame with the C-terminal His-tag provided in the cloning/expression region of the vector. However, this further expression construct (pET22b-GAMT-His-Tag) behaved like the pET22b-GAMT and pET45b-GAMT described above, with an undetectable recombinant GAMT expression (Figure S1E). Thus, unexpectedly, only the N-terminal Tag-based strategies allowed the expression of the recombinant enzyme.

Table S1. Primers used for cloning, site-directed mutagenesis and sequencing.

Fwd, forward; Rev, reverse. †Numbers indicate the 5' position of the sequencing primers, referring to the GAMT coding sequence (NCBI Reference Sequence: NM_000156.5) and to the MAT coding sequence (NCBI Reference Sequence: DQ083239.1). Restriction enzyme cutting sites in cloning primers are underlined. Nucleotide changes in the GAMT mutagenesis primer sequences are in bold and underlined and respective codons are highlighted in grey. Codon changes and relative amino acid substitutions are reported in the third column.

NAME	SEQUENCES (5' to 3')	NOTES
**Cloning primers**		
GAMT PmlI_Fwd	ACGGTACTTCACGTGATGAGCGCCCCCAGCGCGA	
UB-GAMT NcoI_Fwd	ACGGTACTTCCATGGGCCCCCAGCGCGACCCCC	
GAMT HindIII_Rev	GGATACCTAAAGCTTCAGCCTTTGGTCACCAGGGGCG	
MAT2A SacII_Fwd	CGTCTCCGCGGTGGAATGAACGGACAGCTCAACGGC	
MAT2A NotI_Rev	CGTCTGCGGCCGCTCAATATTTAAGCTTTTTGGGCACTTCCC	
**Mutagenesis primers**		**Codon change (aa change)**
UB-GAMT (L37I)	CACGCACCTGCGCATC**A**T**T**GGCAAGCCGGTGATGG	CTG → ATT<br>(Leu→Ile)
UB-GAMT (G38A)	ACCTGCGCATCCTGG**C**CAAGCCGGTGATGGAGC	GGC → GCC<br>(Gly→Ala)
UB-GAMT (L37I - G38A)	CACGCACCTGCGCATC**A**T**T**G**C**CAAGCCGGTGATGGAGC	CTG → ATT<br>(Leu→Ile)<br>GGC → GCC<br>(Gly→Ala)
UB-GAMT (L37M)	ACGCACCTGCGCATC**A**TGGGCAAGCCGGTGATGG	CTG → ATG<br>(Leu→Met)
UB-GAMT (I187Q)	GTACTCAGACATCACC**CAG**ATGTTTGAGGAGACGCAGGTG	ATC → CAG<br>(Ile→Gln)

UB-GAMT (A26N)	GGGCGGCGCCCGCG**AA**CTACGACGCAGCGGAC	GCC → AAC (Ala→Asn)
UB-GAMT (V215Q)	GGAGGTGATGGCGCTG**CAG**CCACCGGCCGACTGCC	GTC → CAG (Val→Gln)
**Sequencing primers**		
GAMT 7[†]_Fwd	GCCCCCAGCGCGACCCCCATCT	
GAMT 312[†]_Fwd	ACGGCAGACACACAAGGTCATCC	
GAMT 711[†]_Rev	TCAGCCTTTGGTCACCAGGGGC	
MAT2A 100[†]_Fwd	GTGACCAAATCAGTGATGCTGTCC	
MAT2A 398[†]_Fwd	AGACCAGGGCTTAATGTTTGGC	
MAT2A 799[†]_Fwd	TTGTGGACACTTATGGCGGTTG	
T7 promoter	TAATACGACTCACTATAGGG	
T7 terminator	GCTAGTTATTGCTCAGCGG	

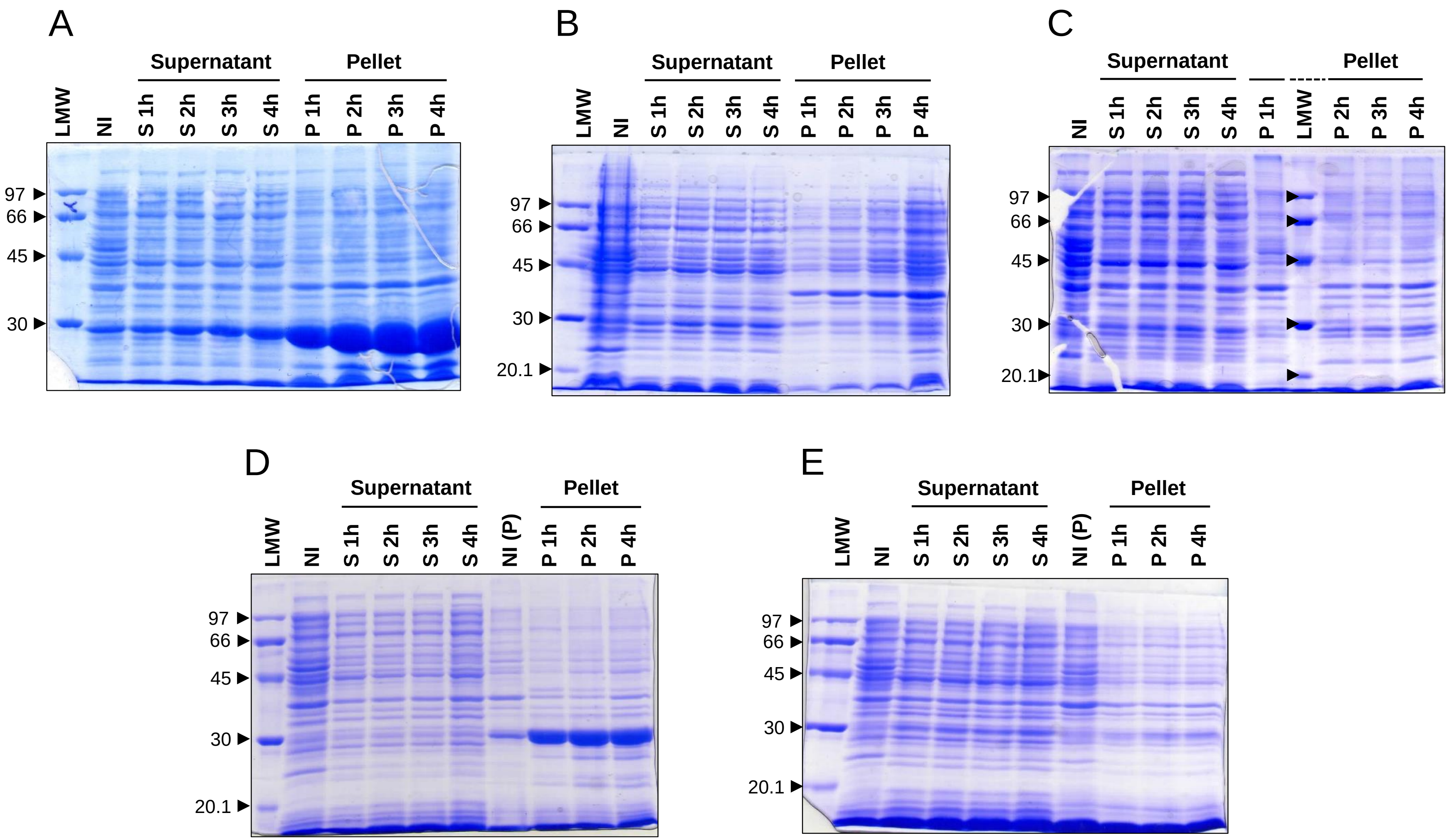

Figure S1: Cloning strategies to optimize recombinant human GAMT expression. [A] pET45b/His-GAMT; [B] pET45b/GAMT (NO tag); [C] pET22b/GAMT (NO tag); [D] pET22b/pelB-GAMT; [E] pET22b/GAMT-His. SDS-PAGE of representative time-course induction experiments with the different expression constructs. Supernatant (S) and Pellet (P) fractions are compared; NI is the not-induced sample. LMW, low molecular weight protein standards.

A

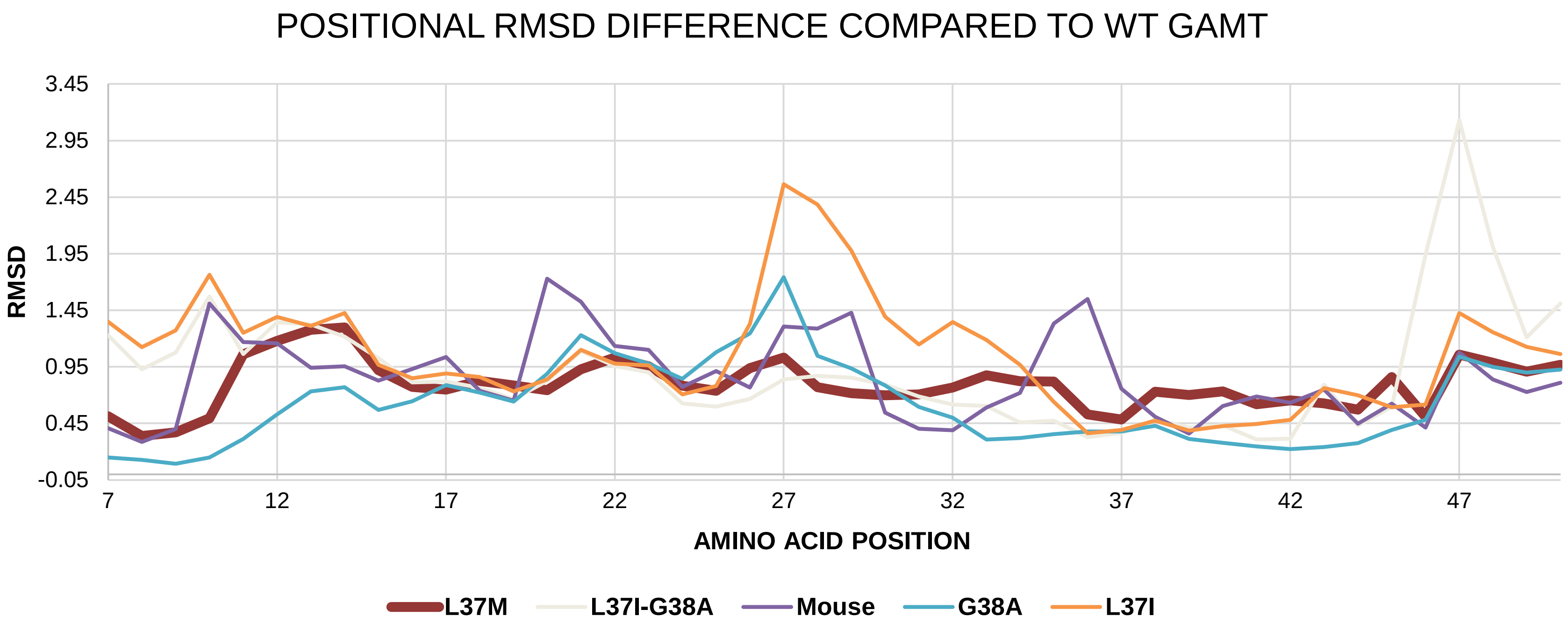

B

	Mean RMSD
**L37I-G38A**	0.93
**L37M**	0.81
**Mouse**	0.88
**G37A**	0.64
**L37I**	1.07

Figure S2: In silico studies of GAMT protein flexibility. [A] Graph plotting the predicted root-mean-square deviation (RMSD) difference of the GAMT mutants compared to wild type, against protein chain position for the first 47 amino acid residues. [B] Mean RMSD difference of the GAMT mutants compared to wild type GAMT for the first 47 amino acid residues.

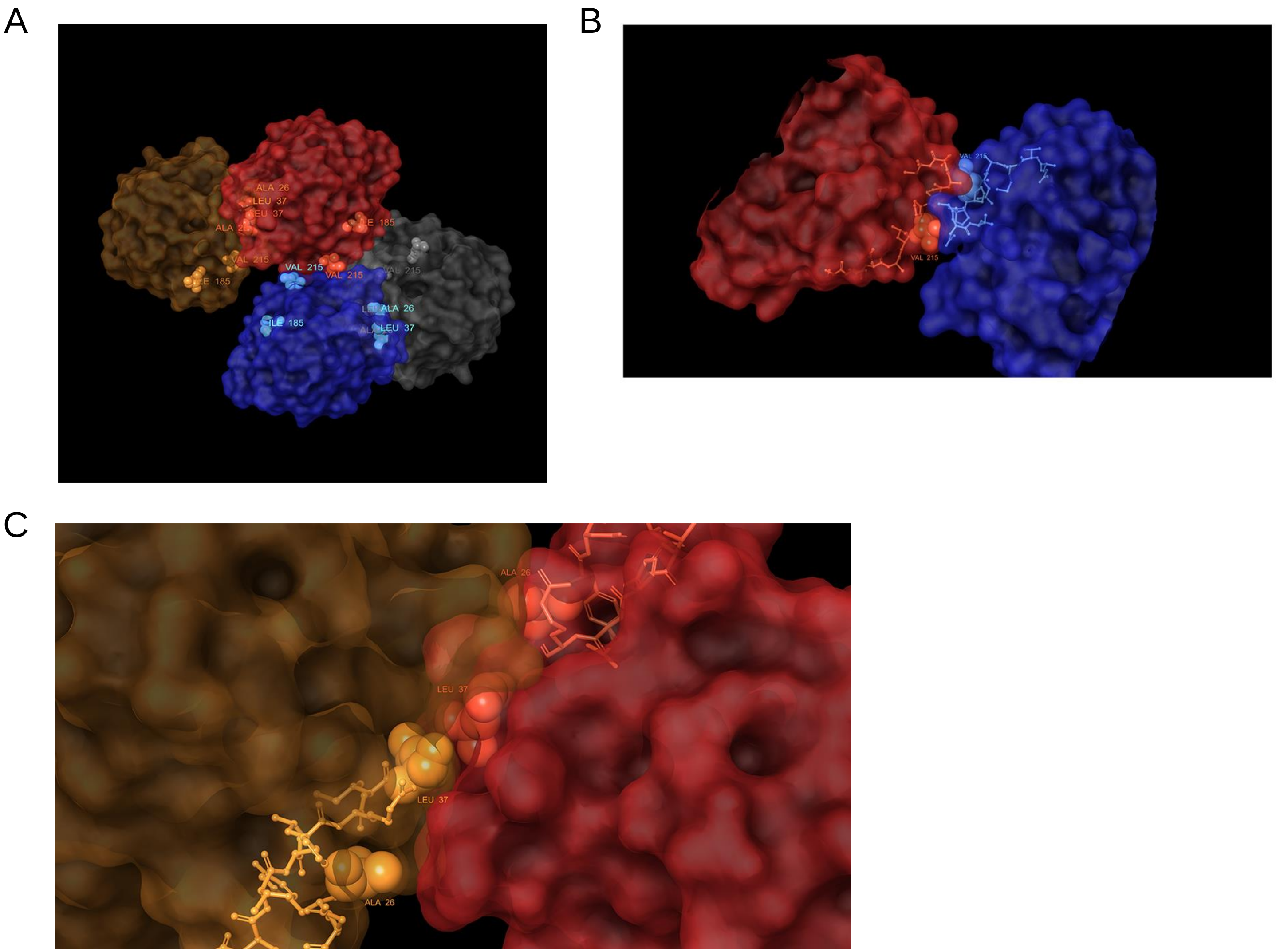

Figure S3: In silico studies of GAMT intermolecular interactions. [A] GAMT homotetramer as in 3orh PDB. The mutagenized amino acid residues are highlighted. [B] GAMT homodimer interface involving Val215 residues. [C] GAMT homodimer interface involving Ala26 and Leu36 residues.

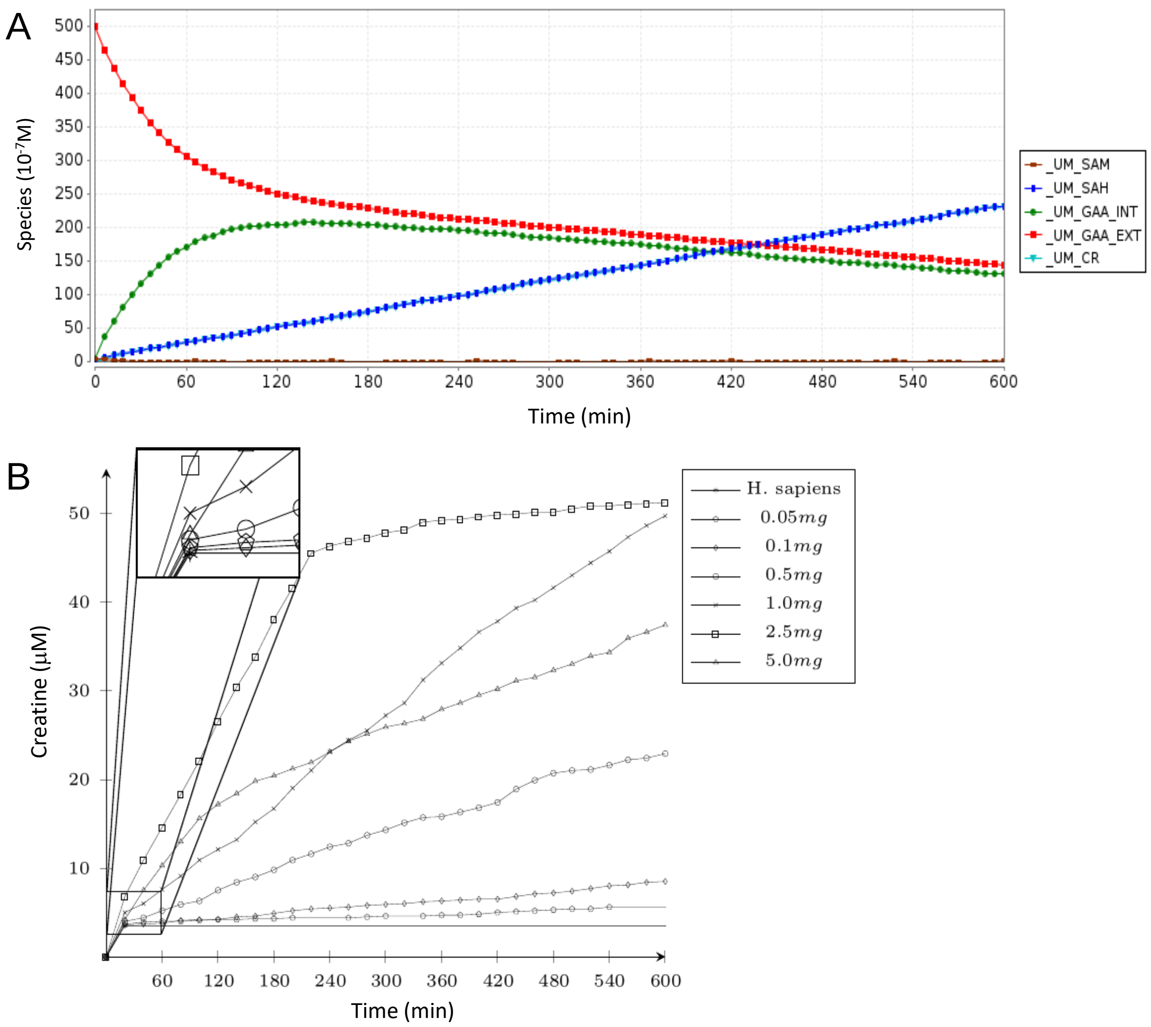

Figure S4: **Metabolic modeling of GAMT-loaded RBCs.** [A] Behaviour of RBCs engineered with 1 mg of recombinant GAMT cloned from *E. coli*. The Y axis indicates the concentration (10^{-7}M) of the different species. [B] Bio-PEPA model of Creatine synthesis pathway by engineered RBCs loaded with a fixed amount of GAMT (1mg) and different amounts of recombinant MAT (as detailed in the inset). Each model was simulated for 10 hours (X axis), with a sampling every 10 minutes.

REFERENCES

Newman, E.B., Budman, L.I., Chan, E.C., Greene, R.C., Lin, R.T., Woldringh, C.L., D'Ari, R. (1998). Lack of S-adenosylmethionine results in a cell division defect in Escherichia coli. J Bacteriol. 180, 3614-3619.
